# Structure Elucidation of the Metabolites of 2', 3', 5'-Tri-*O*-Acetyl-*N*_6_-(3-Hydroxyphenyl) Adenosine in Rat Urine by HPLC-DAD, ESI-MS and Off-Line Microprobe NMR

**DOI:** 10.1371/journal.pone.0127583

**Published:** 2015-06-01

**Authors:** Wei Guo, Mengxia Jin, Zhaoxia Miao, Kai Qu, Xia Liu, Peicheng Zhang, Hailin Qin, Haibo Zhu, Yinghong Wang

**Affiliations:** 1 State Key Laboratory of Bioactive Substance and Function of Natural Medicines & Ministry of Health Key Laboratory of Biosynthesis of Natural Products, Chinese Academy of Medical Sciences and Peking Union Medical College, Beijing, China; 2 Beijing Key Laboratory of New Drug Mechanisms and Pharmacological Evaluation Study, Chinese Academy of Medical Sciences and Peking Union Medical College, Beijing, China; Old Dominion Univ., UNITED STATES

## Abstract

2', 3', 5'-tri-*O*-acetyl-*N_6_*-(3-hydroxyphenyl) adenosine (also known as WS070117) is a new adenosine analog that displays anti-hyperlipidemic activity both *in vitro* and *in vivo* experiments as shown in many preliminary studies. Due to its new structure, little is known about the metabolism of WS070117. Hence, the *in vivo* metabolites of WS070117 in rat urine following oral administration were investigated. Identification of the metabolites was conducted using the combination of high-performance liquid chromatography (HPLC) coupled with diode array detector (DAD), ion trap electrospray ionization-mass spectrometry (ESI-MS), and off-line microprobe nuclear magnetic resonance (NMR) measurements. Seven metabolites were obtained as pure compounds at the sub-milligram to milligram levels. Results of structure elucidation unambiguously revealed that the phase I metabolite, *N_6_*-(3-hydroxyphenyl) adenosine (M8), was a hydrolysate of WS070117 by hydrolysis on the three ester groups. *N_6_*-(3-hydr-oxyphenyl) adenine (M7), also one of the phase I metabolites, was the derivative of M8 by the loss of ribofuranose. In addition to two phase I metabolites, there were five phase II metabolites of WS070117 found in rat urine. 8-hydroxy-*N_6_*-(3-hydroxy-phenyl) adenosine (M6) was the product of M7 by hydrolysis at position 8. The other four were elucidated to be *N_6_*-(3-*O-β*-D-glucuronyphenyl) adenine (M2), *N_8_*-hydroxy-*N_6_*-(3-*O*-sulfophenyl) adenine (M3), *N_6_*-(3-*O-β*-D-glucuronyphenyl) adenosine (M4), and *N_6_*-(3-*O*- sulfophenyl) adenosine (M5). Phase II metabolic pathways were proven to consist of hydroxylation, glucuronidation and sulfation. This study provides new and valuable information on the metabolism of WS070117, and also demonstrates the HPLC/MS/off-line microprobe NMR approach as a robust means for rapid identification of metabolites.

## Introduction

Hyperlipidemia is defined as the presence of abnormally elevated levels of lipids and/or lipoproteins in the plasma, including total cholesterol (TC), triglyceride (TG), low-density lipoprotein Cholesterol (LDL-C) [1]. It is a prevalent disease and a major symptom of the metabolic syndrome resulting from a variety of genetic and environmental factors. This disease increases morbidity and mortality when combined with other prevalent diseases such as diabetes mellitus, hypertension, and cardiovascular diseases, which is estimated to be about 20 million globally in 2015 [2]. Therefore, increasing anti-hyperlipidaemia agents are utilized for the prevention and treatment of hyperlipidaemia. However, most of them possess considerable side effects.

WS070117 [2', 3', 5'-tri-*O*-acetyl-*N*_6_-(3-hydroxyphenyl) adenosine] is a lipid regulator with a new structure discovered by investigators in our institute. Because of its high efficacy and low toxicity, WS070117 is being developed as an anti- hyperlipidaemia agent. WS070117 was shown to improve lipid metabolism disorder and fatty liver in high-fat-diet (HFD) fed Syrian golden hamsters by activating AMP-activated protein kinase (AMPK), an important cellular energy sensor [3,4]. In our current study, we found that WS070117 significantly reduced atherosclerotic plaque formation in apoE^-/-^ mice through promoting high-density lipoprotein (HDL) cholesterol efflux capacity [5]. The possible underlying mechanism was the inhibition of ATP-binding cassette transporter A1 (ABCA1) protein degradation via suppressing calpain activity by WS070117. Our previous studies have demonstrated WS070117 as a promising drug candidate for the treatment of hyperlipidemia and atherosclerosis. A new substance patent has been filed for the chemical entity of WS070117 and its application in the treatment of hyperlipidaemia [6]. Due to its new structure, the systematic metabolic behaviors of WS070117 *in vivo* remain unclear, and whether the WS070117 itself or its metabolites play the lipid lowing role needs to be further investigated. Therefore, characterization of the *in vivo* metabolic pathways of WS070117 is especially important.

Quadrupole time-of-flight mass spectrometry (Q/TOF-MS) significantly con- tributes to the characterization of drug metabolites by providing accurate mass of ions and revealing valuable structural information through the MS^2^ spectra. It is reliable and facilitates the structural determination [7–9]. Meanwhile, the data analysis software Metabolynx can automatically generate lists of expected and unexpected metabolites by comparing the post-acquisition data of the analytes with those of the control [10]. Despite recent advancement of various analytical tools, the metabolite elucidation of compounds undergoing multiple and unpredictable metabolism in the biological matrix continues to present a challenge [11].

Nuclear magnetic resonance (NMR) spectroscopy has been proved to be an app- ropriate analytical method for structure determination of organic compounds, incl- uding stereo chemical information [12,13]. It has played a central role in the understanding of metabolic processes of drugs for more than 30 years [14–16]. Nevertheless, a key disadvantage of NMR technique lies in the fact that it is relatively insensitive, has a lower detection range of 1–5 μM, and requires a relatively large sample size (~500 μl). The two main approaches to increase the sensitivity of NMR include the use of higher field magnets and the development of more sensitive NMR probes. The ultra-high field magnet technology has finally broken the 1 GHz barrier [17], and cryogenically cooled probes have increased the sensitivity by three to four folds [18]. The combination of these two techniques has advanced the art of NMR to the point where microgram quantities can be sufficient for structure elucidations. Although LC-DAD-MS-SPE/cryoNMR hyphenation has been proven to be a valuable tool to isolate and analyze small quantities of metabolites directly from biological fluids [19–21], the ultra-high field magnet and hyphenation are expensive and uncommon. In addition, the complexity to install cryogenically cooled probes often can limit the versatility of the NMR platform. An alternative approach is to use small value NMR probes or micro-coil technology to increase sensitivity [18,22]. The conventional NMR sampling mode still consists of a cylindrical glass tube of 5-mm outer diameter and 20-cm length with a solution volume of 500–600 μL, whereas for small value NMR probes, the sample volumes are down to the microliter range. Micro-NMR probes of 1.7 mm to 1mm have also been developed by Bruker with sample volumes being 50 to 2.5 μL [23–25]. It has been shown that the mass sensitivity (sensitivity of the probe to receive signals from a defined number of nuclei) of the 10 μL micro-coil probe is approximately ten times greater than a conventional NMR probe [26]. Micro-coil or microbore NMR probes have been used for more than 15 years [27], and substantial improvements in their design, heteronuclear capabilities, availability, and sensitivity now allow the analysis of nanomoles of materials (as low as 5 nL in volumes) [27]. Using a single glass capillary tube for each individual sample is an alternative to the flow injection method in several areas of analytical NMR, such as the structural characterization of mass- and volume-limited samples, the coupling of microbore HPLC to NMR, and applications where very high sample throughput (HT NMR) is essential but only a limited sample volume is available. Meanwhile, the use of micro-probe provides a more convenient way to measure and quantify biological samples with a very small volume/weight/cell count, including new trace natural products from plants [27,28].

In the present study, 7 metabolites of WS070117 were observed in rat urine from the HPLC trace. There were peaks not only for 1D ^1^H, COSY, HSQC, and HMBC, but also for ^13^C, determined from DEPT NMR spectra using a Bruker 1.7 PA TXImicroprobe (active volume 30 μL). Unambiguous identification of the metabolites was completed using on-line HPLC-DAD, MS and off-line NMR. In addition, the principal metabolic pathways of WS070117 in rats were proposed for the first time for the understanding of *in vivo* metabolic process of WS070117.

## Materials and Methods

### Ethics Statement

All of the protocols using in this study were approved by The Animal Care & Welfare Committee Institute of Materia Medica, Chinese Academy of Medical Sciences and Peking Union Medical College (License Number: 00000079). The care of laboratory animal and the animal experimental operations were performed in accordance with Beijing Administration Rule of Laboratory Animal. We confirm this study is approved by The Animal Care & Welfare Committee Institute of Materia Medica, Chinese Academy of Medical Sciences and Peking Union Medical College (License Number: 00000079).

### Chemicals and reagents

WS070117 was provided by Prof. Wu Song from Institute of Materia Medica, Chinese Academy of Medical Sciences & Peking Union Medical College, with purity of 99.4% as assessed by HPLC. Methanol was obtained from Merck (Darmstadt, Germany). Pure water was prepared using a Milli-Q water purification system (Millipore Corporation, Billerica, MA, USA). The sample was prepared by dissolving WS070117 powder (5 g) in aqueous 0.5% carboxymethyl cellulose sodium (50 ml), and stirring to disperse uniformly. The sample was stored at 4°C for less than 3 days.

### Animal dosing and sample collection

Ten male Sprague-Dawley rats (weighing about 200 g each) were obtained from Vital River Laboratory Animal Technology Co. Ltd., Beijing, China (License Number: SCXK Beijing 2012–0001). All protocols complied with the National Institutes of Health regulations for the care and use of animals in research. Throughout the acclimatization and study periods, all animals had access to corresponding food and water ad libitum and were maintained on a 12 h light/dark cycle (21±2°C with a relative humidity of 45±10%). Rats were acclimatized for 7 days in cages prior to model construction. Animals were then randomly allocated into two groups, control (n = 2) and administrated (n = 8). The dosing volume was determined by the weight of animals and was about 2 ml per animal. Animals in the administrated group were treated with WS070117 once daily by oral gavage for 1 week, while the rats in the control group were administrated with 0.5% carboxymethyl cellulose sodium. Urine samples were collected in ice-cold tubes for 24 h after the last dosing, and then all rats were sacrificed by cervical dislocation. Pooled urine of control and administrated group was respectively centrifuged at 12000 rpm for 10 min, and the supernatants were then collected. Urine samples were freeze-dried and stored at -80°C for further analysis.

### HPLC analysis of urine metabolites

Chromatography was performed on a Prominence LC-20A chromatography system (Shimadzu) consisting of two LC-20AD and one LC-20AB pump modules, a CBM-20A control unit, a SPD-M20A DAD, a SIL-20AC Auto sampler and a FRC-10A fraction collector. Compared with the conventional ultraviolet detector (UVD), DAD can provide ultraviolet spectra and chromatographic spectra at the same time by full wavelength scanning. Hence DAD has multiple significant advantages in separating complex biological samples, including distinguishing chromatographic peaks, identifying the purity of chromatographic peaks, selecting the best wavelength for examining and editing the wavelength program for mixture analysis [29,30]. In the present study, we utilized DAD to select the best wavelength of WS070117 at 299 nm. The dried urine powders were dissolved in methanol by a dilution factor of 10. Separations were performed on a RP C_18_ column (4.6×250 mm, 5 μm, Shimadzu). The flow rate was 1ml/min. The mobile phase gradient program (methanol/water) was 1:99 (t = 0 min), 1:99 (t = 10 min), 10:90 (t = 25 min), 60:40 (t = 55 min), 100:0 (t = 60 min), 100:0 (t = 65 min), 1:99 (t = 66 min) and 1:99 (t = 71 min) for WS070117. It was 1:99 (t = 0 min), 1:99 (t = 10 min), 10:90 (t = 25 min), 60:40 (t = 55 min), 1:99 (t = 55.5 min), and 1:99 (t = 61 min) for the metabolites of WS070117. The injection volume was 20 μL. Metabolite constituents were fractionated on the same HPLC system for NMR measurement. A number of separations were conducted, and the constituents were enriched in fraction collectors, then dried by nitrogen. These samples were stored at -20°C prior to NMR analysis.

### MS analysis of urine metabolites

All compounds were obtained by preparative HPLC method and diluted to about 0.1 ng/μL in methanol. The HRESI-MS spectra were acquired using an AccuTOF CS mass spectrometer (JMS-T100CS, JEOL, Tokyo, Japan). The needle voltage was set at 2000 V; the orifice 1 voltage was set at 60 V; the orifice 2 voltage was 20 V; and the flow rate and temperature of spray gas were 2.5 L/min and 250°C, respectively. The MS/MS experiments were carried out on a QTRAP 5500 mass spectrometer (AB Sciex, Foster City, USA). The mass spectrometer was equipped with an electrospray ionization source in positive ion mode with temperature maintained at 450°C. Other parameters were set as follows: curtain gas (CUR) 20; ion spray (IS) voltage 4500; nebulizer gas 1 (GS1) 50; nebulizer gas 2 (GS2) 40; decluster potential (DP) 40; entrance potential (EP) 10. The collision energy (CE) was 25, 25, 25, 32, 32, 42, 42 and 25 for WS070117, M2, M3, M4, M5, M6, M7 and M8, respectively.

### NMR analysis of urine metabolites

All NMR experiments were recorded at 25°C on a Bruker AVANCE-III 500 NMR spectrometer (^1^H, 500.06 MHz; ^13^C, 125.75 MHz) equipped with 1.7 PA TXImicroprobe. Standard pulse sequences from Topspin 3.0 software package (Bruker BioSpin) were used. Sample was dissolved in DMSO-d_6_ and centrifuged at 3000 g for 5 min at 298 K, and 60 μl sample was then transferred into a 1.7-mm NMR tube. Chemical shifts were given on the scale and referenced to the residual solvent peak of DMSO-d_6_ at 2.49 and 39.5 ppm for proton and carbon, respectively. Coupling constants (J) were given in hertz. The pulse conditions were as follows: For the ^1^H NMR spectra: spectrometer frequency (SF) 500.063 MHz, acquisition time (AQ) 3.172 s, relaxation delay (RD) 1.000 s, pulse 90° spectral width (SW) 10330.578 Hz, FT size 64 K data. For the ^13^C NMR spectra: SF 125.753 MHz, AQ 1.101 s, RD 1.000 s, pulse 56° SW 29761.904 Hz, FT size 32 K data. For the HSQC spectra: AQ 0.1321 s, RD 1.000 s, SW 4504.504 Hz (^1^H) and 25149.912 Hz (^13^C). A sinebell function was applied to the F2 dimension before zero filled to 2 K points, and a sinebell function was applied to the F1 dimension and zero filled to 2 K points before Fourier transformation. A one-bond coupling constant of 145.0 Hz and a long-range coupling constant of 8.0 Hz were used to set delays in the pulse sequence. For the HMBC spectra: AQ 0.5284 s, RD 0.779 s, SW 5000.000 Hz (^1^H) and 30181.266 Hz (^13^C). A one-bond coupling constant of 145.0 Hz and a long-range coupling constant of 8.0 Hz were used to set delays in the pulse sequence. A sinebell weighting was applied to both ^1^H and ^13^C dimensions and zero filled to 4 and 1 K points. For the COSY spectra: AQ 0.2642 s, RD 0.889 s, SW 4347.826 Hz. A sinbell weighting was applied to each dimension and zero filled to 2 K points.

## Results and Discussion

**Fig 1:**
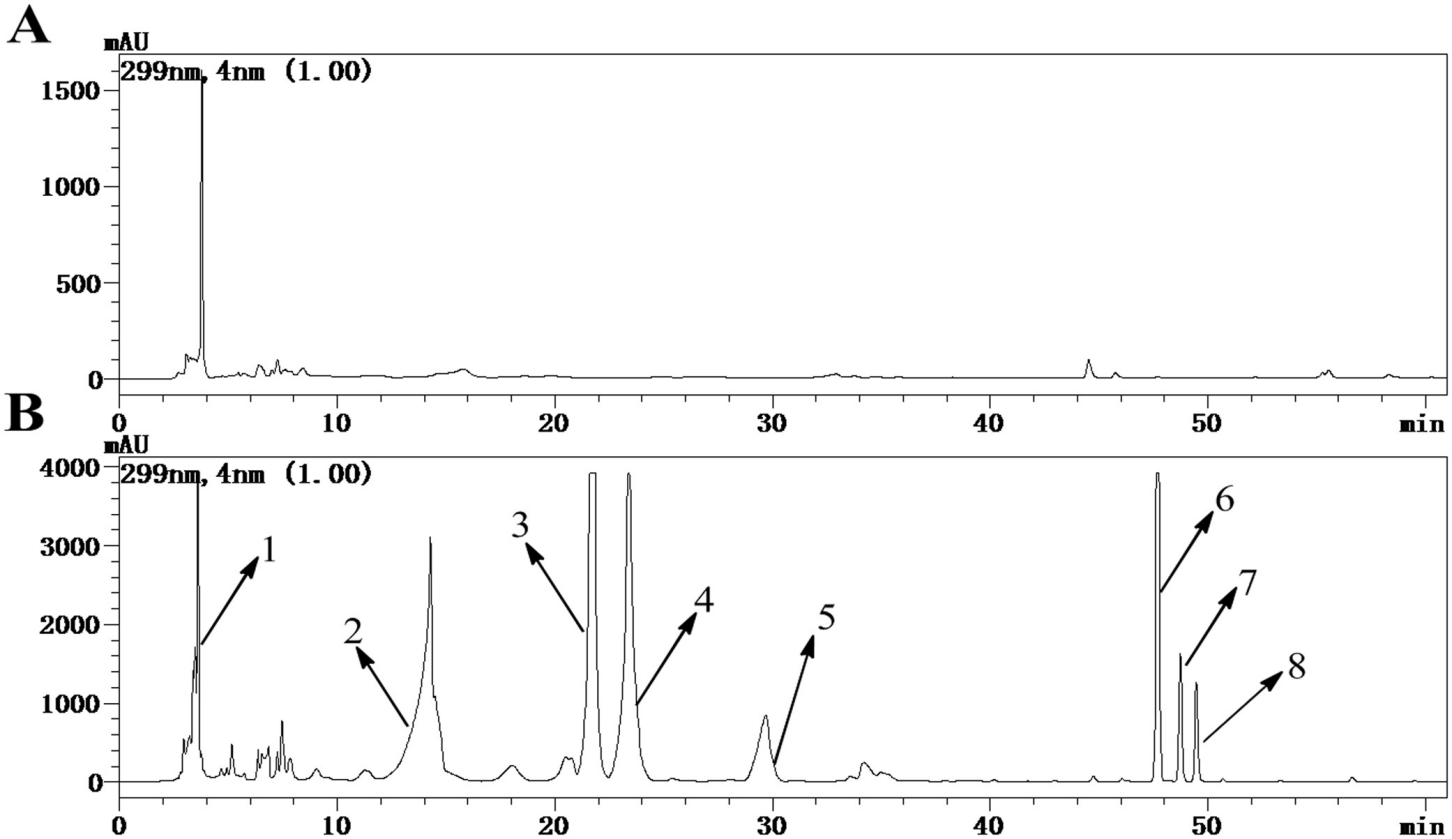
HPLC chromatogram of WS010117 metabolites in urine of control (A) and administrated (B) rats at 299 nm. Peak 1: 3.60 min (M1); 2: 14.15 min (M2); 3: 21.58 min (M3); 4: 23.25 min (M4); 5: 29.48 min (M5); 6: 47.68 min (M6); 7: 48.73 min (M7); 8: 49.44 min (M8).

**Fig 2:**
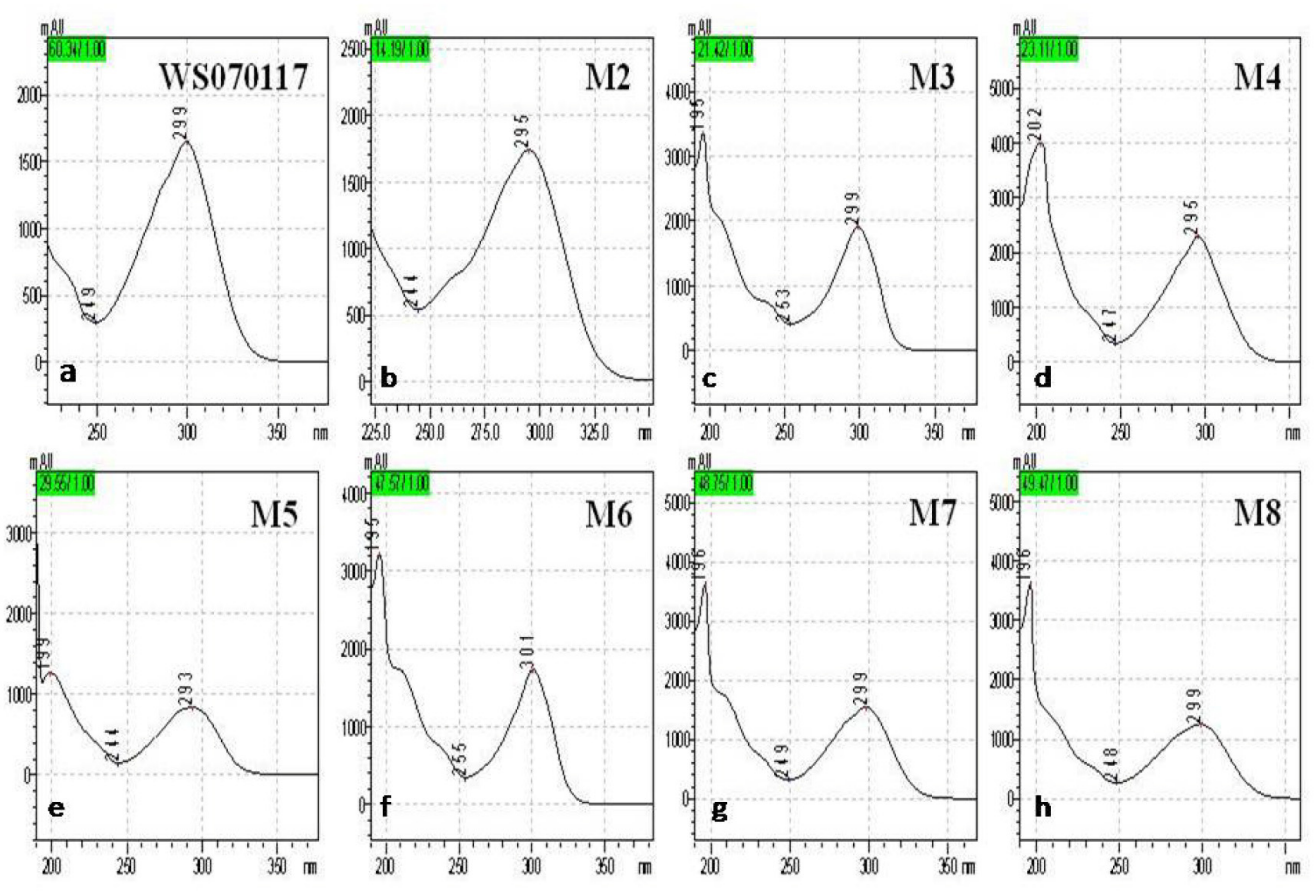
UV spectra of (a) WS070117; (b) M2; (c) M3; (d) M4; (e) M5; (f) M6; (g) M7; and (h) M8.

A gradient-based reversed-phase HPLC procedure was developed for the analysis of urine samples from rats orally receiving 1g/kg of WS010117, enabling the observation of 8 well-resolved peaks at 299 nm of UV (DAD) detector. The administrated group has 7 peaks more than the control (Fig 1). The separated components M2-M8 shared a similar type of UV spectrum to WS070117 with one maximal absorption band recorded at about 299 nm (Fig 2), suggesting the existence of adenine. The UV spectrum of M1 was very different from that of WS070117 and represented a very complex mixture, which was demonstrated by 1D ^1^H NMR spectrum. HRESI-MS and ESI-MS^2^ were performed in the positive ion ESI mode on all metabolites. The former afforded their accurate masses and molecular formulas, while the latter provided the fragmentation patterns and the corresponding masses of fragment ions (Table 1). For qualitative NMR measurement, metabolites M2-M8 were isolated and enriched at sub-milligram to milligram levels. Unambiguous structure identifications were based on in-depth 1D and 2D NMR analysis for selected peaks. All the carbons of M4, M5, M6 and M8 were assigned according to their 1D ^13^C NMR and DEPT spectra (Table 2), and the ^13^C NMR data of M2, M3, and M7 were provided by their heteronuclear correlations spectra (HSQC and HMBC).

**Table 1:** The retention time, predicted elemental compositions, observed mass and calculated mass, characteristic fragment ions, and description of metabolites of WS070117 in rat urine.

Metabolites	Retention time (min)	Elemental compositions	Measured mass [M+H]^+^ (m/z)	Calculated mass [M+H]^+^ (m/z)	Error (mmu)	Error (ppm)	Fragment ions	Metabolite description
M2	14.15	C17H17N5O7	404.12172	404.12062	1.10	2.72	228	Glucuronidation
M3	21.58	C11H9N5O5S	324.04148	324.04026	1.22	3.75	278, 244, 233, 189	Sulfonation
M4	23.25	C22H25N5O11	536.16300	536.16288	0.12	0.22	404, 360, 281, 228, 147	Glucuronidation
M5	29.48	C16H17N5O8S	440.08961	440.08761	2.00	4.58	360, 308, 228	Sulfonation
M6	47.68	C11H9N5O2	244.08192	244.08345	-1.53	-6.25	227, 135, 109	Hydroxylmetabolite of M7
M7	48.73	C11H9N5O	228.08764	228.08853	-0.89	-3.92	135, 109	Losing ribofuranosemetabolite of M8
M8	49.44	C16H17N5O5	360.13059	360.13079	-0.21	-0.57	228, 163	Hydroxylmetabolite of WS070117

**Table 2:** Structure assignments of WS070117 and its metabolites with ^1^H, 13C and HMBC NMR data from off-line measurements.

metabolite	Peak^a^	^1^H NMR date^b^ (δ, ppm; *J*, Hz)^c^	^13^C NMR date^b^ (δ, ppm)	HMBC ^1^H to ^13^C^c^
2', 3', 5'-tri-*O*-acetyl-*N*_6_-(3-hydroxylaniline) adenosine	WS070117	δ 9.83 (s, 1H, 6-NH), 9.33 (s, 1H, 3'-OH), 8.52 (s, 1H, 8-H), 8.42 (s, 1H, 2-H), 7.49 (dd, *J* = 2.3, 2.3 Hz, 1H, 2''-H), 7.31 (dd, *J* = 8.1, 2.3 Hz, 1H, 4''-H), 7.08 (dd, *J* = 8.1, 8.1 Hz, 1H, 5''-H), 6.46 (dd, *J* = 8.1, 2.3 Hz, 1H, 6''-H), 6.27 (d, *J* = 5.2 Hz, 1H, 1'-H), 6.06 (dd, *J* = 5.6, 5.2 Hz, 1H, 2'-H), 5.65 (dd, *J* = 5.6, 3.2 Hz, 1H, 3'-H), 4.43 (dd, *J* = 11.7, 3.7 Hz, 1H, 5'-Ha), 4.39 (m, 1H, 4'-H), 4.25 (dd, *J* = 11.7, 5.3 Hz, 1H, 5'-Hb), 2.12 (s, 3H, 3'-CH3), 2.04 (s, 3H, 2'-CH3), 2.01 (s, 3H, 5'-CH3).	δ170.01 (5'-C = O), 169.43 (3'-C = O), 169.27 (2'-C = O), 157.36 (3''-C), 152.28 (2-C), 152.21 (6-C), 149.12 (4-C), 140.86 (8-C), 140.38 (1''-C), 128.94 (5''-C), 120.27 (5-C), 111.87 (6''-C), 110.03 (4''-C), 108.11 (2''-C), 85.75 (1'-C), 79.43 (4'-C), 71.99 (2'-C), 70.00 (3'-C), 62.74 (5'-C), 20.46 (5'O-CH3), 20.35 (3'O-CH3), 20.19 (2'O-CH3).	(2,4), (2,6), (8,4), (8,5), (8,1'), (1',2'), (1',4), (1',8), (2',1'), (2',4), (2',2'-C = O), (3',1,), (3',5'), (3',3'-C = O), (4',3'), (4',3'), (5',3'),(5',4'), (5',5'-C = O), (2'',1''), (2'',3''), (2'',4''), (2'',6''), (4'',2''), (4'',3''), (4'',6''), (5'',1''), (5'',2''), (5'',3''), (5'',4''), (5'',6''), (6'',1''), (6'',2''), (6'',4''), (6'',5''), (6-NH,1''), (6-NH,5), (6-NH,6), (6-NH,2''), (6-NH,6''), (3-OH,1''), (3-OH,2''), (3-OH,3'') (3-OH,4''), (2'-CO-CH3, 2'-CO), (3'-CO-CH3,3'-CO), (5'-CO-CH3,5'-CO).
*N*_6_-(3-*β*-D-glucuroniyl-laniline) purin	M2	δ 8.33 (s, 1H, 2-H), 8.16 (s, 1H, 8-H), 7.64 (dd, *J* = 2.3, 2.3 Hz, 1H, 2''-H), 7.54 (dd, *J* = 8.2, 2.3 Hz, 1H, 4''-H), 7.21 (dd, *J* = 8.2, 8.2 Hz, 1H, 5''-H), 6.71 (dd, *J* = 8.2, 2.3 Hz, 1H, 6''-H), 4.82 (d, *J* = 7.5 Hz, 1H, *β*-glu-H), 3.57 (m, 1H, 4'''-H), 3.44 (m, 1H, 5'''-H), 3.285 (m, 1H, 3'''-H), 3.23 (m, 1H, 2'''-H)	δ 172.78 (6'''-C = O), δ157.98 (3''-C), 150.12 (2-C), 143.50 (8-C), 152.86 (4-C), 151.39 (6-C), 141.35 (1''-C), 129.46 (5''-C), 113.90 (4''-C), 108. 68 (6''-C), 118.23 (5-C), 109.78 (2''-C), 100.23 (1'''-C), 75.86 (3'''-C), 74.33 (4'''-C), 65.31 (5'''-C), 73.37 (2'''-C).^d^	(2,4), (2,6), (8,4), (8,5), (2'',1''), (2'',3''), (2'',4''), (2'',6''), (4'',2''), (4'',3''), (4'',6''), (5'',1''), (5'',3''), (5'',6''), (6'',2''), (6'',4''), (β-glu-H,3'').
*N*_8_-hydroxy-*N*_6_-(3- Sulfo-lailine) purin	M3	δ 8.87 (s, 6-NH or 3''-OH), 8.17 (s, 1H, 2-H), 7.61 (dd, *J* = 8.2, 2.3 Hz, 1H, 6''-H), 7.49 (dd, *J* = 2.3, 2.2 Hz, 1H, 2''-H), 7.18 (dd, *J* = 8.2, 8.2 Hz, 1H, 5''-H), 6.77 (dd, *J* = 8.2, 2.2 Hz, 1H, 4''-H).	δ 153.76 (3''-C), 154.43 (2-C), 150.20 (8-C), 149.09 (4-C), 142.48 (6-C), 141.35 (1''-C), 128.95 (5''-C), 114.36 (4''-C), 114.05 (6''-C), 111.38 (2''-C), 106.26 (5-C).^d^	(8,4), (8,5), (8,1'), (2'',1''), (2'',3''), (2'',4''), (2'',6''), (4'',2''), (4'',3''), (4'',6''), (5'',1''), (5'',2''), (5'',3''), (5'',4''), (5'',6''), (6'',1''), (6'',2''), (6'',4'').
*N*_6_-(3-*β*-D-glucuronide) adenosine	M4	δ 9.90 (s, 6-NH), 8.52 (s, 1H, 8-H), 8.41 (s, 1H, 2-H), 7.75 (dd, *J* = 2.3, 2.3 Hz, 1H, 2''-H), 7.58 (dd, *J* = 8.2, 2.3 Hz, 1H, 6''-H), 7.21 (dd, *J* = 8.2, 8.2 Hz, 1H, 5''-H), 6.76 (dd, *J* = 8.2 2.3 Hz, 1H, 4''-H), 5.98 (d, *J* = 5.9 Hz, 1H, 1'-H), 4.83 (d, *J* = 7.3 Hz, 1H, β-glu-H), 4.62 (dd, *J* = 5.9, 5.5 Hz, 1H, 2'-H), 4.17 (dd, *J* = 5.5, 4.1 Hz, 1H, 3'-H), 3.97 (m, 1H, 4'-H), 3.69 (br d, *J* = 12.4 Hz, 1H, 5'-Ha), 3.57 (br d, *J* = 12.4 Hz, 1H, 5'-H), 3.42 (m, 1H, 5'''-H), 3.26 (m, 1H, 3'''-H), 3.23 (m, 1H, 2'''-H), 3.18 (m, 1H, 4'''-H).	δ172.95 (6'''-C = O), 158.05 (3''-C), 152.47 (2,6-C), 149.61 (4-C), 141.21 (8-C), 140.76 (1''-C), 129.73 (5''-C), 120.68 (5-C), 114.61 (6''-C), 111.08 (4''-C), 109.28 (2''-C), 100.69 (β-glu-C), 88.42 (1'-C), 86.28 (4'-C), 76.89 (3'''-H), 74.68 (5'''-H), 74.11 (2'-C), 73.44 (2'''-H), 72.41 (6'''-H), 70.95 (3'-C), 61.96 (5'-C).	(2,4), (2,5), (2,6), (8,4), (8,5), (1',2'), (1',4), (1',8), (2',1'), (2',4'), (3',1,), (3',4), (3',5'), (4',1'), (4',2'), (4',3'), (4',5'), (5',3'),(5',4'), (2'',1''), (2'',3''), (2'',4''), (2'',6''), (4'',2''), (4'',3''), (4'',6''), (5'',1''), (5'',3''), (5'',4''), (5'',6''), (6'',1''), (6'',2''), (6'',4''), (β-glu-H,3''), (6-NH,5), (6-NH,6), (6-NH,2''), (6-NH,6'').
*N*_6_-(phenol-3-Sulfo-lailine) adenosine	M5	δ 9.91 (s, 6-NH), 8.52 (s, 1H, 8-H), 8.40 (s, 1H, 2-H), 7.74 (dd, *J* = 2.3, 2.3 Hz, 1H, 2''-H), 7.63 (dd, *J* = 8.4, 2.3 Hz, 1H, 6''-H), 7.20 (dd, *J* = 8.5, 8.5 Hz, 1H,5''-H), 6.97 (dd, *J* = 8.5, 2.3 Hz, 1H, 4''-H), 5.95 (d, *J* = 5.9 Hz, 1H, 1'-H), 4.69 (dd, *J* = 5.9, 5.4 Hz, 1H, 2'-H), 4.17 (dd, *J* = 5.4, 3.6 Hz, 1H, 3'-H), 3.97 (m, 1H, 4'-H), 3.67 (br d, *J* = 11.9 Hz, 1H, 5'-Ha), 3.57 (br d, *J* = 11.9 Hz, 1H, 5'-Hb).	δ 153.62 (3''-C), 152.34 (6-C), 152.25 (2-C), 149.51 (4-C), 140.98 (8-C), 140.15 (1''-C), 128.75 (5''-C), 120.54 (5-C), 116.25 (6''-C), 115.59 (4''-C), 113.78 (2''-C), 88.20 (1'-C), 86.10 (4'-C), 73.90 (2'-C), 70.78 (3'-C), 61.79 (5'-C).	(2,4), (2,6), (8,4), (8,5), (8,1'), (1',2'), (1',4), (1',8), (2',1'), (2',4), (3',1,), (3',5'), (4',3'), (4',3'), (5',3'),(5',4'), (2'',3''), (4'',2''), (4'',3''), (4'',6''), (5'',1''), (5'',3''), (5'',4''), (5'',6''), (6'',1''), (6'',2''), (6'',4''), (6-NH,5), (6-NH,6), (6-NH,2''), (6-NH,6'').
*N*_8_-hydroxy-*N*_6_- (3-hydroxylaniline) purin	M6	δ 9.00 (s, 6-NH or 3''-OH), 8.13 (s, 1H, 2-H), 7.34 (dd, *J* = 2.0, 2.2 Hz, 1H, 2''-H), 7.11 (dd, *J* = 8.0, 2.2 Hz, 1H, 6''-H), 7.05 (dd, *J* = 8.0, 8.0 Hz, 1H, 5''-H), 6.39 (dd, *J* = 8.0, 2.2 Hz 1H, 4''-H).	δ 157.66 (3''-C), 154.43(2-C), 149.54 (8-C), 149.25 (4-C), 142.60 (6-C), 141.35 (1''-C), 129.29 (5''-C), 110.12 (6''-C), 108.97 (4''-C), 107.29 (5-C), 106.31 (2''-C).	(8,4), (8,5), (8,1'), (2'',1''), (2'',3''), (2'',4''), (2'',6''), (4'',2''), (4'',3''), (4'',6''), (5'',1''), (5'',2''), (5'',3''), (5'',4''), (5'',6''), (6'',2''), (6'',4'').
*N*_6_-(3-hydroxylaniline) purin	M7	δ 9.44 (s, 6-NH or 3''-OH), 8.29 (s, 1H, 2-H), 8.10 (s, 1H, 8-H), 7.57 (dd, *J* = 2.2, 2.2 Hz, 1H, 2''-H), 7.30 (dd, *J* = 8.1 2.2 Hz, 1H, 6''-H), 7.05 (dd, *J* = 8.1, 8.1 Hz, 1H, 5''-H), 6.38 (dd, *J* = 8.1, 2.2 Hz, 1H, 4''-H).	δ 157.38 (3''-C), 154.94 (4-C), 150.59 (6-C), 150.12 (2-C), 143.50 (8-C), 141.35 (1''-C), 128.98 (5''-C), 118.97 (5-C), 110.81 (6''-C), 108.86 (4''-C), 106.75 (2''-C).^d^	(2,4), (2,6), (8,4), (8,5), (8,6), (2'',1''), (2'',3''), (2'',4''), (2'',6''), (4'',2''), (4'',3''), (4'',6''), (5'',1''), (5'',3''), (5'',4''), (5'',6''), (6'',2''), (6'',4'').
*N*_6_-(3-hydroxylaniline) adenosine	M8	δ 9.81 (s, 6-NH or 3'-OH), 8.53 (s, 1H, 8-H), 8.40 (s, 1H, 2-H), 7.51 (dd, *J* = 2.2, 2.2 Hz, 1H, 2''-H), 7.31 (dd, *J* = 8.1, 2.2 Hz, 1H, 4''-H), 7.09 (dd, *J* = 8.1, 8.1 Hz, 1H, 5''-H), 6.46 (dd, *J* = 8.1, 2.2 Hz 1H, 6''-H), 5.95 (d, *J* = 6.1 Hz, 1H, 1'-H), 4.64 (dd, *J* = 6.1, 5.5 Hz, 1H, 2'-H), 4.18 (dd, *J* = 5.5, 3.3 Hz, 1H, 3'-H), 3.99 (m, 1H, 4'-H), 3.70 (dd, *J* = 12.1, 3.7 Hz, 1H, 5'-Ha), 3.58 (dd, *J* = 12.1, 3.8 Hz, 1H, 5'-Hb).	δ157.55 (3''-C), 152.26 (6-C), 152.00 (2-C), 149.33 (4-C), 140.73 (8-C), 140.59 (1''-C), 129.10 (5''-C), 120.45 (5-C), 111.84 6 (''-C), 110.10 (4''-C), 108.14 (2''-C), 87.96 (1'-C), 85.96 (4'-C), 73.71 (2'-C), 70.65 (3'-C), 61.66 (5'-C).	(2,4), (2,6), (8,4), (8,5), (8,1'), (1',2'), (1',4), (1',8), (2',1'), (2',4'), (3',1,), (3',5'), (4',3'), (4',3'), (5',3'),(5',4'), (2'',1''), (2'',3''), (2'',4''), (2'',6''), (4'',2''), (4'',3''), (4'',6''), (5'',1''), (5'',2''), (5'',3''), (5'',4''), (5'',6''), (6'',1''), (6'',2''), (6'',4''), (6'',5'').

^a^ Retention times with method used for HPLC-PDA analysis.

^b 1^H (500 MHz), ^13^C (125 MHz) and HMBC NMR spectral data measured in DMSO-d_6_.

^c^Multiplicity of signals: s, singlet; d, doublet; t, triplet; m, multiplet; br, broad. Coupling constants (apparent splittings) reported as numerical values in hertz.

^d^ Obtained from HSQC and HMBC spectra.

### ^1^H NMR and ESI-MS^2^ spectral characteristics of WS070117

**Fig 3:**
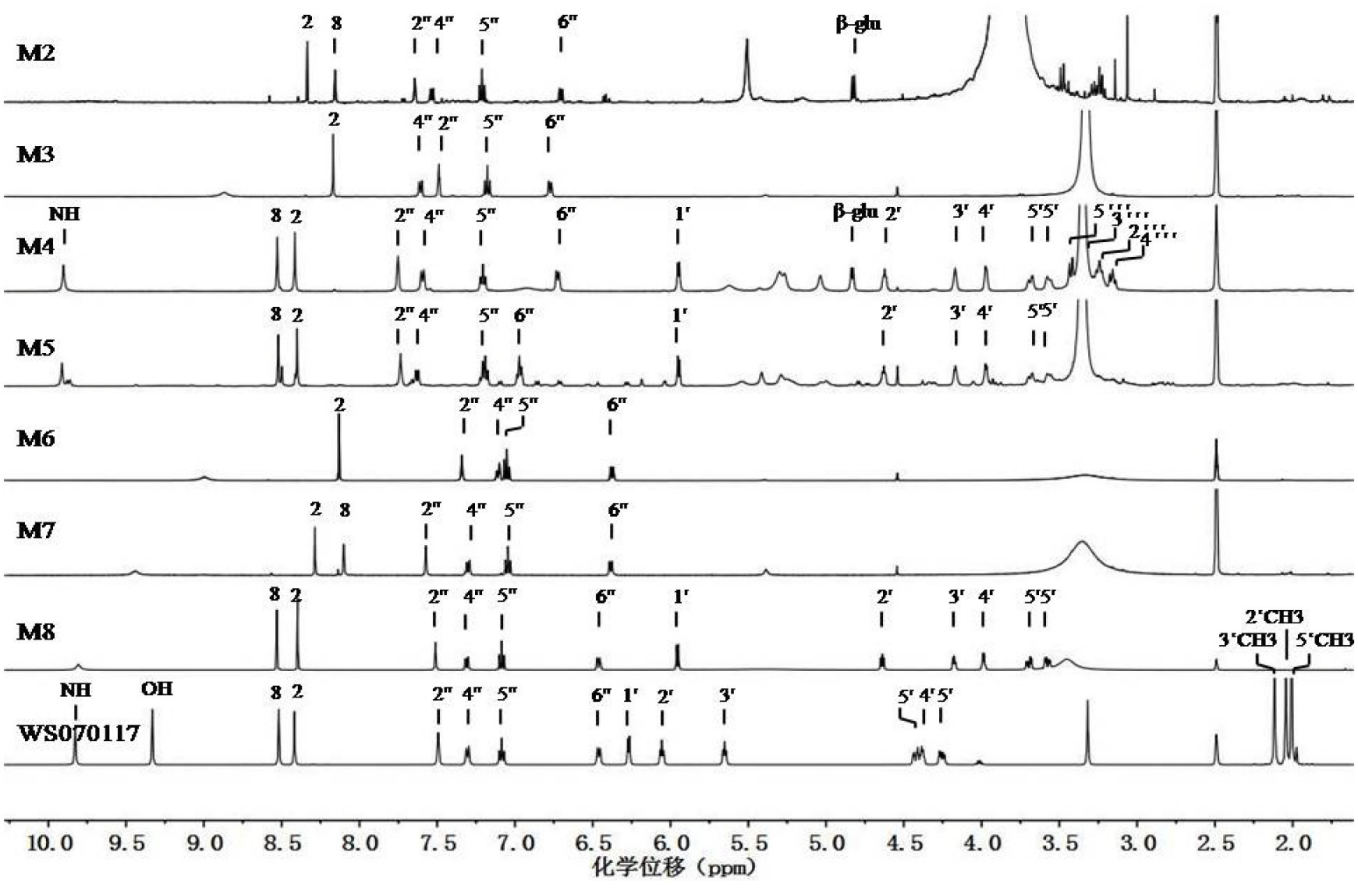
The stack of ^1^H NMR spectra of WS010117 and its seven metabolites (in DMSO-d_6_).

**Fig 4:**
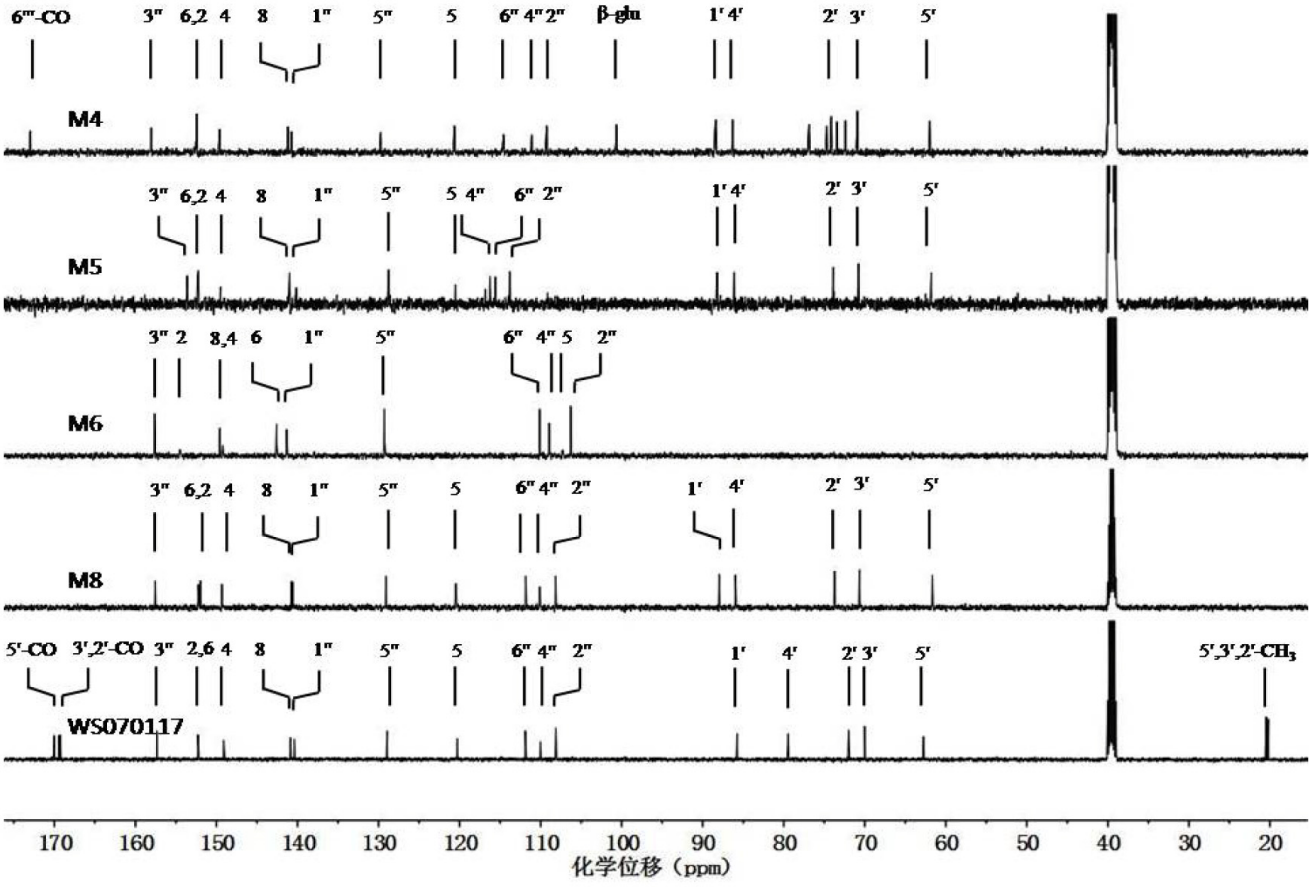
The stack of ^13^C NMR spectra of WS010117 and its four metabolites (in DMSO-d_6_).

WS070117 is a derivative of adenosine prepared through structure modification, with three hydroxyls at C-2', 3', and 5' on adenosine acetylized, and 6-NH_2_ on adenosine substituted by 3-hydroxyphenyl. The ESI-MS of WS070117 afforded an [M+H]^+^ ion at *m/z* 486, and the molecular formula for WS070117 was C_22_H_23_N_5_O_8_ (S1 File). Fragmentation of this precursor ion yielded the fragment ions at both *m/z* 259 and 228. The ^1^H NMR spectrum of WS070117 (Fig 3: WS070117) was identical to the above structural features with the adenosine moiety being identified by the signals at δ 9.83 (1H, s, 6-NH), 8.52 (1H, s, 8-H), 8.42 (s, 1H, 2-H), 6.27 (d, *J* = 5.2 Hz, 1H, 1'-H), 6.06 (dd, *J* = 5.6, 5.2 Hz, 1H, 2'-H), 5.65 (dd, *J* = 5.6, 3.2 Hz, 1H, 3'-H), 4.43 (dd, *J* = 11.7, 3.7 Hz, 1H, 5'-Ha), 4.39 (m, 1H, 4'-H) and 4.25 (dd, *J* = 11.7, 5.3 Hz, 1H, 5'-Hb), consistent with the literature values [31]. The three acetyl groups and 3-hydroxyphenyl were demonstrated at δ 2.12 (s, 3H, 3'-OCOCH_3_), 2.04 (s, 3H, 2'-OCOCH_3_), 2.01 (s, 3H, 5'-OCOCH_3_), 7.49 (dd, *J* = 2.3, 2.3 Hz, 1H, 2''-H), 7.31 (dd, *J* = 8.1, 2.3 Hz, 1H, 4''-H), 7.08 (dd, *J* = 8.1, 8.1 Hz, 1H, 5''-H), 6.46 (dd, *J* = 8.1, 2.3 Hz, 1H, 6''-H) and 9.33 (1H, s, 3'-OH). The ^13^C and DEPT NMR and HSQC spectra (Table 2, Fig 4: WS070117, S1 File) also exactly displayed characteristic carbons of three acetyls, one ribofuranosyl, one adenine, and one 3-hydroxyphenyl. Complete assignments of the ^1^H and ^13^C NMR data were made by in-depth analysis of the HMBC spectrum (S1 File), which showed the following long range ^1^H-^13^C correlative information H-2' to 2'-carbonyl; H-3' to 3'-carbonyl; H-5' to 5'-carbonyl; H-8 to C-4, 5, and 1'; H-2 to C-4 and 6; and NH-6 to C-6, 1'', 2'', and 6''.

### Structural determination of M8, M4, and M5

HRMS of M8 (Table 1) afforded a pseudomolecular ion peak at *m/z* 360.1306 [M+H]^+^, suggesting a molecular formula of C_16_H_17_N_5_O_5_. A comparison of the ^13^C NMR, DEPT, and HSQC spectra (Table 2, Fig 4: M8, WS070117, S1 and S8 Files) of M8 to those of WS070117 revealed carbons in M8 due to the presence of one ribofuranosyl, one adenine, and one 3-hydroxyphenyl. ^1^H NMR spectrum further indicated that M8 closely resembled WS070117. The main difference between M8 and WS070117 was that M8 lacked the signals from three acetyl groups of WS070117 but displayed the deacetylization shift of H-2' (-1.42), H-3' (-1.47), and H-5' (-0.63) in the ^1^H NMR spectrum and of C-2' (-1.72), C-3' (-0.65), and C-5' (-1.08) in the ^13^C NMR spectrum, respectively. Furthermore, ESI-MS showed the pseudomolecular ion peak at *m/z* 360 [M+H]^+^ (Table 1), which was 126 amu less than that of WS070117, again indicating the loss of three acetyl groups in M8. Complete assignments of ^1^H and ^13^C NMR signals of M8 were performed by detailed 1D and 2D NMR experiments (mainly HMBC experiments, see S8 File). Therefore, M8 was determined as *N*_6_-(3-hydroxylaniline) adenosine.

HRMS of M4 gave a pseudomolecular ion peak at *m/z* 536.1625 [M+H]^+^, not only indicating that the molecular formula of M4 was C_22_H_25_N_5_O_11_, but also showing that M4 was 176 amu higher than that of M8 (Table 1: M4, M8). The ESI-MS^2^ of the pseudomolecular ion peak gave a fragment ion peak at m/z 360, confirming the above result. A comparison of the ^1^H and ^13^C NMR spectra of M4 to those of M8 revealed their close resemblance (Fig 3: M4, M8 and Fig 4: M4, M8). The main difference between M4 and M8 was the appearance of a glucuronyl group in M4, with its signals being observed at δ 4.83 (d, *J* = 7.2 Hz, 1H, 1'''-H), 3.42 (m, 1H, 5'''-H), 3.26 (m, 1H, 3'''-H), 3.23 (m, 1H, 2'''-H) and 3.18 (m, 1H, 4'''-H) in the ^1^H NMR spectrum; and at δ 100.69 (1'''-C), 76.89 (3'''-C), 74.68 (5'''-C), 73.44 (2'''-C) and 72.41 (4'''-C) in the ^13^C NMR spectrum, respectively. The coupling constant of the anomeric proton was in the range of 7–8 Hz which allowed its *β*-configurationto be assigned [32]. The glucuronyl group was confirmed by extensive explorations on the 1D and 2D NMR experiments. The ^1^H-^1^H COSY experiment established the spin system from 1'''-H to 5'''-H in glucuronyl group, and the HMBC experiment (S4 File) showed the long range ^1^H-^13^C correlations from H-8 to C-4, 5 and 1'; H-2 to C-4 and 6; NH-6 to C-6, 1'', 2'' and 6''. The long range ^1^H-^13^C correlation in HMBC from H-1''' to C-3'' strongly indicated that the linkage of glucuronyl group and aniline between C-3'' and C-1''' was through ether bond. Thus M4 was determined as *N*_6_-(3-*O*-*β*-D-glucuronyphenyl) adenosine.

As determined by HRMS at m/z 440.0896, the molecular formula for M5 was C_16_H_17_N_5_O_8_S (Table 1: M5). ESI-MS^2^ of the pseudomolecular ion peak at m/z 440 [M+H]^+^ yielded the characteristic ion peaks at *m/z* 360 [M+H-HSO_3_]^+^ and 308 [M+H-ribofuranose-3-OH]^+^. A comparison of the ^1^H and ^13^C NMR spectra of M5 to those of M8 (Table 2, Fig 3: M5, M8 and Fig 4: M5, M8) revealed their close resemblance. The main difference was the downfield chemical shift from signals of the 3-substituted phenyl moiety, with the ^1^H NMR resonances at δ 7.74 (dd, *J* = 2.3, 2.3 Hz, 1H, 2''-H), 7.63 (dd, *J* = 8.4, 2.3 Hz, 1H, 6''-H), 7.20 (dd, *J* = 8.5, 8.5 Hz, 1H, 5''-H), and 6.97 (dd, *J* = 8.5, 2.3 Hz, 1H, 4''-H); and the ^13^C NMR signals appearing at δ 153.62 (3''-C), 140.15 (1''-C), 128.75 (5''-C), 116.25 (6''-C), 115.59 (4''-C), and 113.78 (2''-C), respectively. Based on the aforementioned molecular formula for M5 and the absence of 3''-OH signal in the ^1^H NMR spectrum, a hydrogensulfated 3-hydroxylphenyl moiety was unambiguously established. Thus M5 was identified as *N*_6_-(3-*O*-sulfophenyl) adenosine.

### Structural determination of M7 and M2

HRMS of M7 gave a pseudomolecular ion peak at m/z 228.0885 [M+H]^+^, which was 132 amu less than that of M8 (Table 1: M7, M8), indicating a molecular formula of C_11_H_9_N_5_O as well as the loss of ribofuranosyl group from M8. A detailed comparison between M7 and M8 indicated that M7 showed the almost consistent ^1^H and ^13^C NMR spectroscopic data with those of the *N*_6_-(3-hydroxyphenyl) adeninemoiety of M8, but the signals of ribofuranose in M8 were completely disappeared in M7 (Table 2, Fig 3: M7, M8 and Fig 4: M8, S7 File). In addition, the ^13^C NMR spectrum showed the deglycosidation shifts of C-4 (+5.61) and C-8 (+2.75), indicating that the ribofuranosyl group at N-9 of M8 was substituted by hydrogen atom. Thus, M7 was elucidated to be *N*_6_-(3-hydroxylphenyl) adenine.

HRMS of M2 gave a pseudomolecular ion peak at m/z 404.1217 [M+H]^+^, which was 132 amuless than that of M4 (Table 1: M2, M4), indicating a molecular formula of C_17_H_17_N_5_O_7_ as well as the loss of ribofuranose from M4. A detailed comparison between M2 and M4 (Table 2, Fig 3: M2, M4 and Fig 4: M4, S2 File) showed that M2 exhibited the consistent ^1^H and ^13^C NMR spectroscopic data with those of the *N*_6_-(3-*O*-*β*-D-glucuronyphenyl) adenine moiety of M4, but the signals of ribofuranose in M4 were completely disappeared in M2. In addition, the ^13^C NMR spectrum showed the deglycosidation shifts of C-4 (+3.25) and C-8 (+2.29), indicating that the ribofuranosyl group at N-9 of M4 was substituted by hydrogen atom. Thus, M2 was identified to be *N*_6_-(3-*O*-*β*-D-glucuronyphenyl) adenine.

### Structural determination of M6 and M3

HRMS of M6 gave a pseudomolecular ion peak at m/z 244.0819 [M+H]^+^, indicating a molecular formula of C_11_H_9_N_5_O_2_, which contained one more oxygen atom than M7 (Table 1: M6, M7). The ^1^H NMR spectrum of M6 (Table 2, Fig 3: M6) revealed four aromatic signals from 3-hydroxylaniline moiety at δ 7.34 (dd, *J* = 2.0, 2.2 Hz, 1H, 2''-H), 7.11 (dd, *J* = 8.0, 2.2 Hz, 1H, 6''-H), 7.05 (dd, *J* = 8.0, 8.0 Hz, 1H, 5''-H) and 6.39 (dd, *J* = 8.0, 2.2 Hz, 1H, 4''-H) and one adenine proton at δ 8.13 (s, 1H, 2-H), very similar to that of M7 (Table 2, Fig 3: M7) except for the loss of one signal from the adenine moiety. In addition, the ^13^C NMR spectrum of M6 was very similar to that of M7 (Table 2, Fig 4: M6, S7 File), and their main difference was the downfield chemical shifting of +2.77 in M6 for the carbon signal at δ 149.54 (8-C) compared with its counterpart from adenine moiety of M7. Combined with the molecular formula of M6, a hydroxyl group was determined to be linked to 8-C. This result was confirmed by the long range ^1^H-^13^C correlations from 8.13 (s, 1H, 2-H) to δ 149.25 (4-C) and 142.60 (6-C) in the HMBC spectrum (Table 2 and S6 File), and the absent of the H-8 signal in the ^1^H NMR spectrum. Thus, M6 was established as 8-hydroxy-*N*_6_-(3-hydroxyphenyl) adenosine.

**Fig 5:**
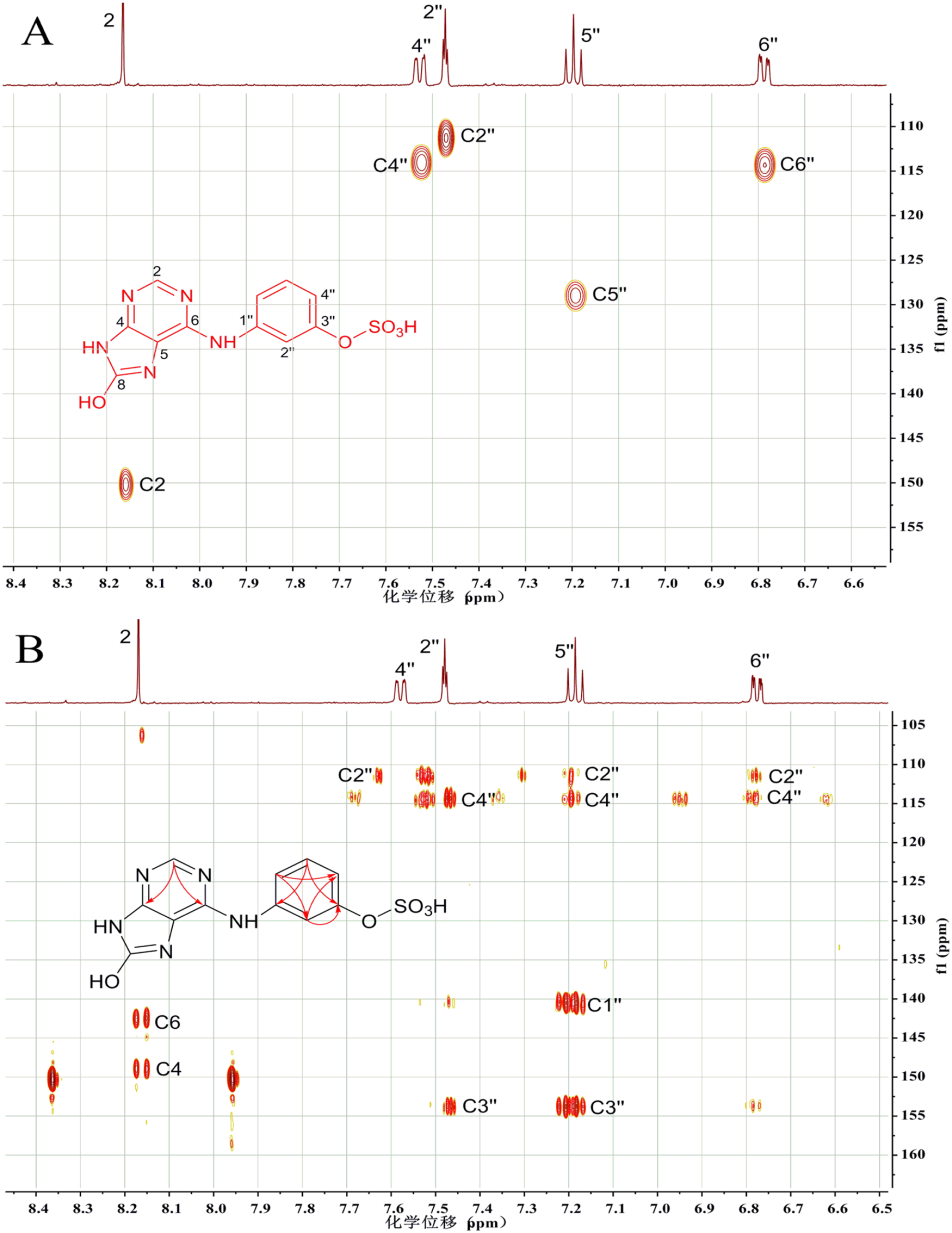
^1^H NMR derived HSQC (A) and HMBC (B) spectra of *N*_8_-hydroxy-*N*_6_-(3-*O*-sulfophenyl) adenine (structure see formula insert). **A** secondary metabolite in rat urine following WS070117 oral administration. The NMR spectra were obtained in deuterated DMSO on a 500 MHz NMR spectrometer, equipped with a 1.7 PA TXI microprobe. (A) HSQC (acquisition time: 2 h): red cross-peaks are stemming from CH, CH_2_ and CH_3_ protons. (B) HMBC (acquisition time: 6 h): the correlation information derived from the marked cross-peaks is summarized in the formula insert.

M3 had a molecular formula of C_11_H_9_N_5_O_5_S by its HRMS at m/z 324.0415. ESI-MS^2^ of the pseudomolecular ion peak at m/z 324 [M+H]^+^ yielded the characteristic ion peak at m/z 244 [M+H–HSO_3_]^+^ (Table 1: M3). The ^13^C NMR data of M3 were provided by their heteronuclear correlations spectra (Fig 5). A comparison of the ^1^H and ^13^C NMR data of M3 to those of M6 (Table 2, Fig 3: M3, M6 and Fig 4: M6, S3 File) revealed their close resemblance. The main difference was the downfield chemical shifting from the 3-substituted aniline moiety, with the esterification shifts in the ^1^H NMR spectrum of H-2'' (+0.15) and H-4'' (+0.38) being observed. Correspondingly, in the ^13^C NMR spectrum, the esterification shifts of C-2'' (+5.07), C-3'' (-3.90), and C-4'' (+5.39) were also obtained. Based on the above molecular formula of M3 and the absence of 3''-OH signal in the ^1^H NMR spectrum, a hydrogensul fated 3-hydroxylaniline moiety was unambiguously established. Thus M3 was identified as *N*_8_-hydroxy-*N*_6_-(3-*O*-sulfophenyl) adenine.

## Conclusion

**Fig 6:**
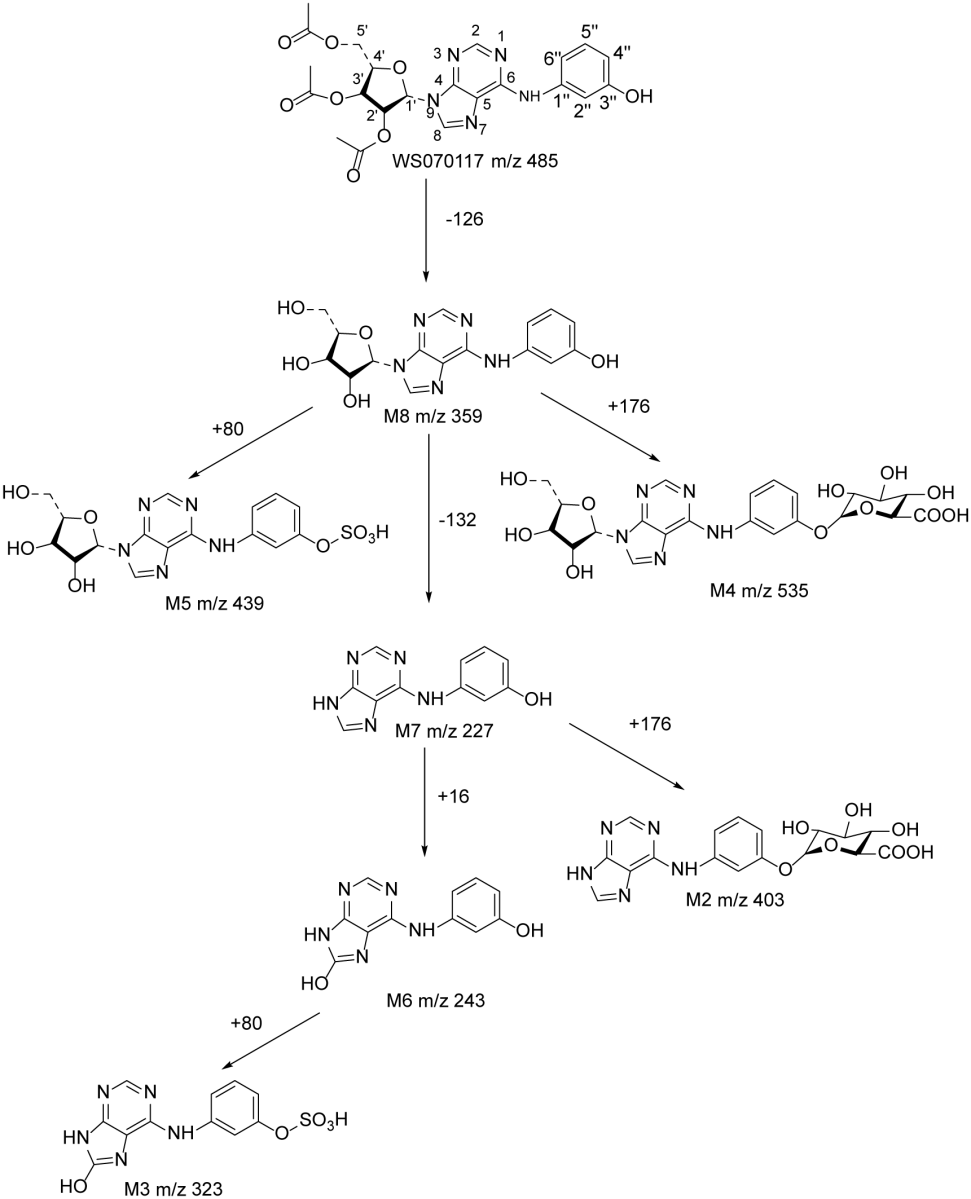
Structures of WS070117 metabolites in rat urine and the proposed metabolic pathways.

In this paper, we have separated seven major metabolites of WS070117 from rat urine and elucidated their structures by mass spectrum and NMR spectroscopy. The metabolic pathways were also proposed as depicted in Fig 6. These results are important for understanding the metabolism of WS070117 in rats, and may provide information for further investigation of the metabolism and excretion of WS070117 in human. In our study, microliter probe played an extremely important and irreplaceable role. The high-resolution 1.7-mm microliter probe with z-gradient allows the measurement of 1D ^1^H, ^13^C NMR and 2D inverse heteronuclear NMR with nanomoles (corresponding to micrograms) of compounds. This method offers significant advantages for the structure elucidation of volume-limited samples, the off-line coupling of HPLC and microprobe NMR, and applications when HT NMR is essential and only limited sample volumes are available. From a more general perspective, this study demonstrates the potential of HPLC/MS/off-line micro-NMR as a robust and effective tool for a rapid chemical characterization of metabolites.

## Supporting Information

S1 File The MS and NMR spectra of WS010117. (PDF)

S2 File The NMR spectra of M2. (PDF)

S3 File The NMR spectra of M3. (PDF)

S4 File The NMR spectra of M4. (PDF)

S5 File The NMR spectra of M5. (PDF)

S6 File The NMR spectra of M6. (PDF)

S7 File The NMR spectra of M7. (PDF)

S8 File The NMR spectra of M8. (PDF)
