# Supplementary material for: Structure Elucidation of the Metabolites of 2', 3', 5'-Tri-O-Acetyl-N 6-(3-Hydroxyphenyl) Adenosine in Rat Urine by HPLC-DAD, ESI-MS and Off-Line Microprobe NMR
Source: PLoS One. 2015 Jun 1;10(6):e0127583. doi: 10.1371/journal.pone.0127583 (PMC4451981; doi:10.1371/journal.pone.0127583)

S1 File. The MS and NMR spectra of WS010117.

Fig. S1-1 Mass spectrum of WS010117 obtained by ESI-MS. (A) EMS spectrum. (B) MS^2 spectrum

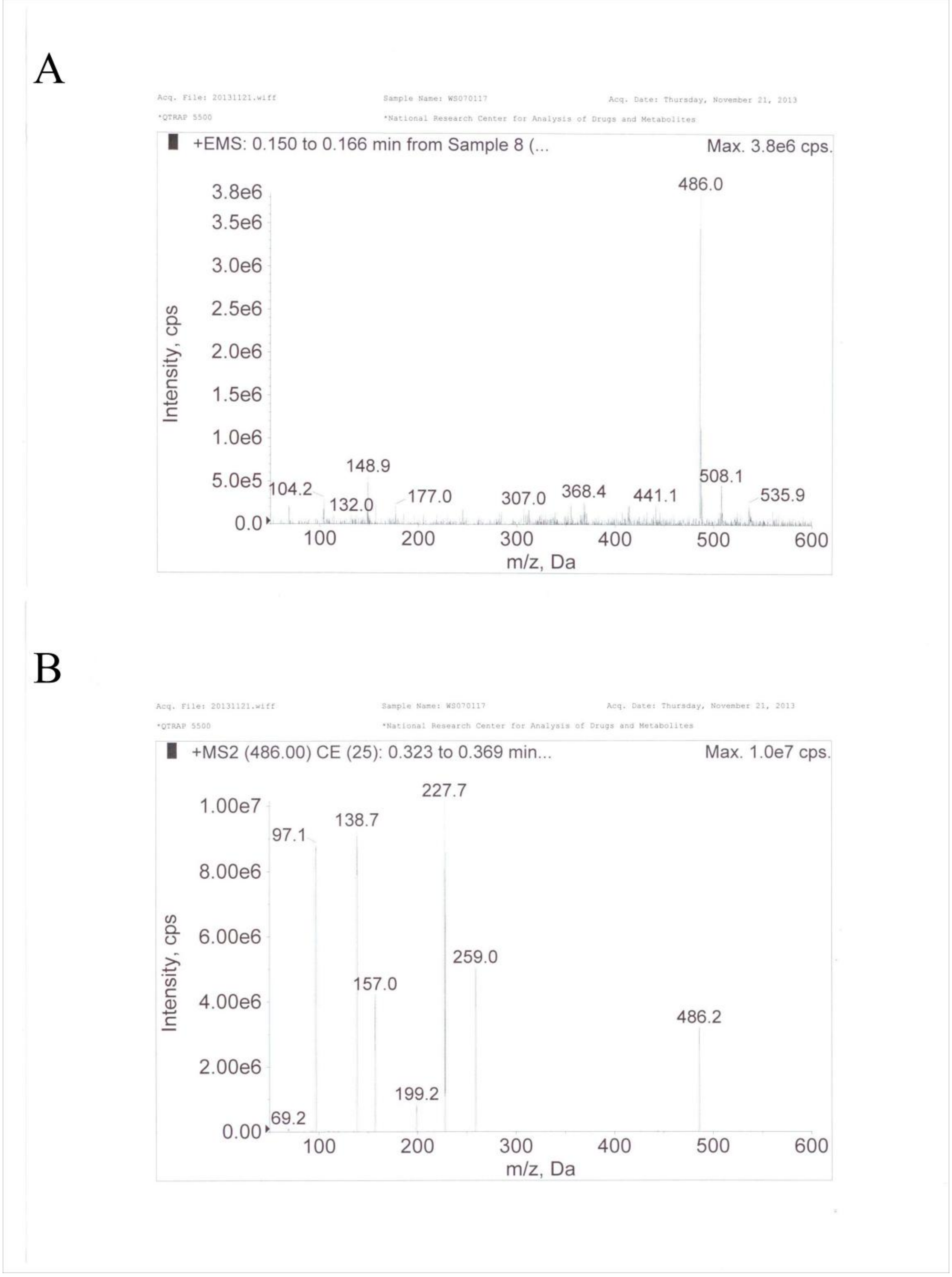

Fig. S1-2 ^{1}H NMR spectrum of WS010117 (500 MHz, DMSO, 25 ℃).

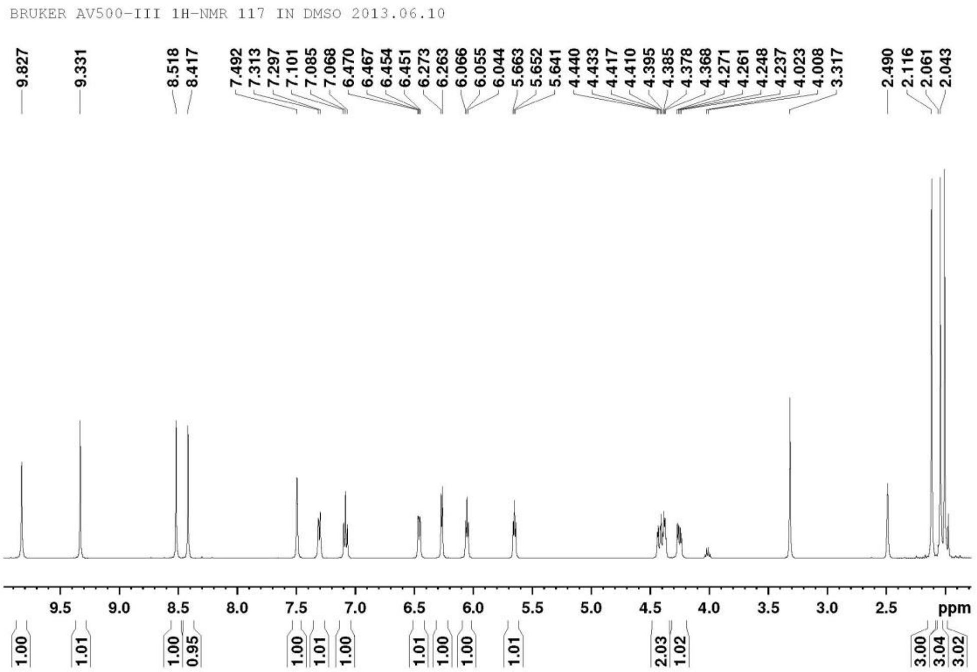

Fig. S1-3 ^{13}C NMR spectrum of WS010117 (500 MHz, DMSO, 25 ℃).

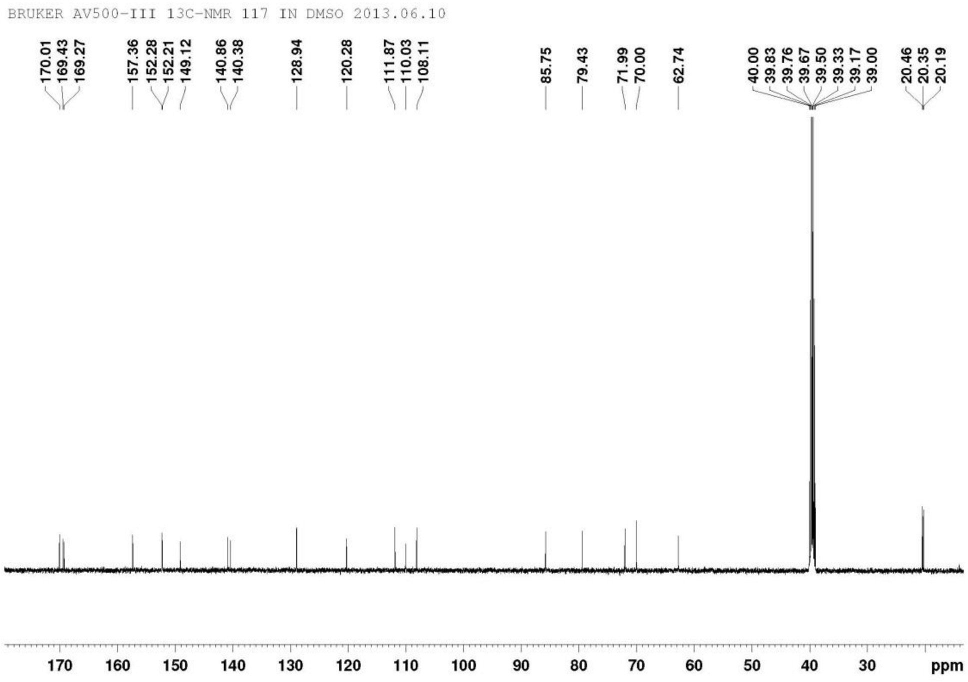

Fig. S1-4 DEPT NMR spectrum of WS010117 (500 MHz, DMSO, 25 ℃).

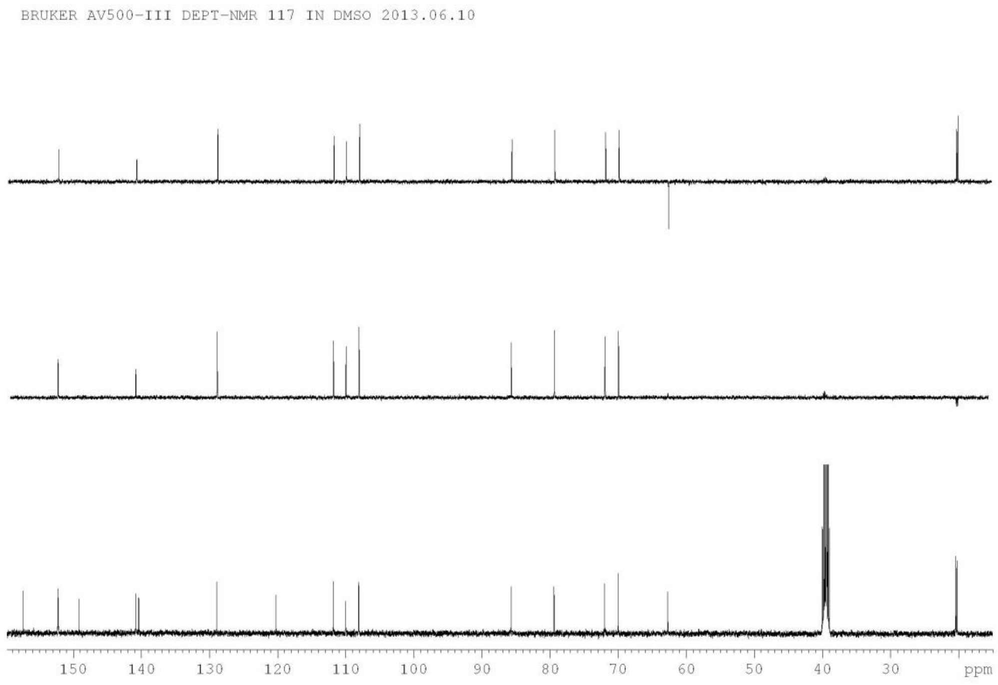

Fig. S1-5 COSY NMR spectrum of WS010117 (500 MHz, DMSO, 25 ℃).

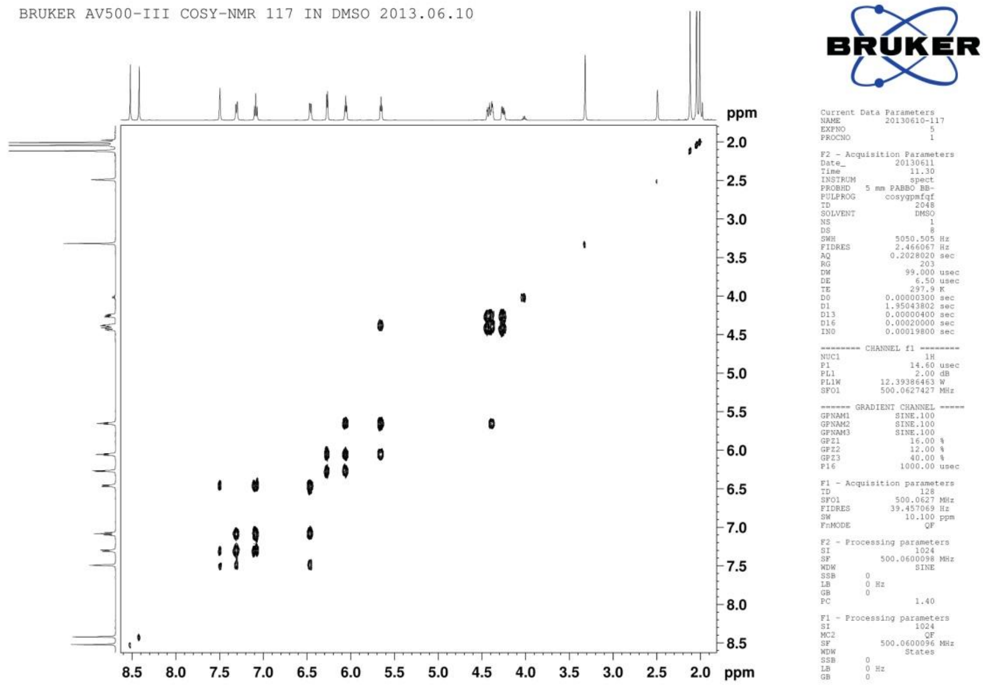

Fig. S1-6 COSY NMR spectrum of WS010117 (500 MHz, DMSO, 25 ℃).

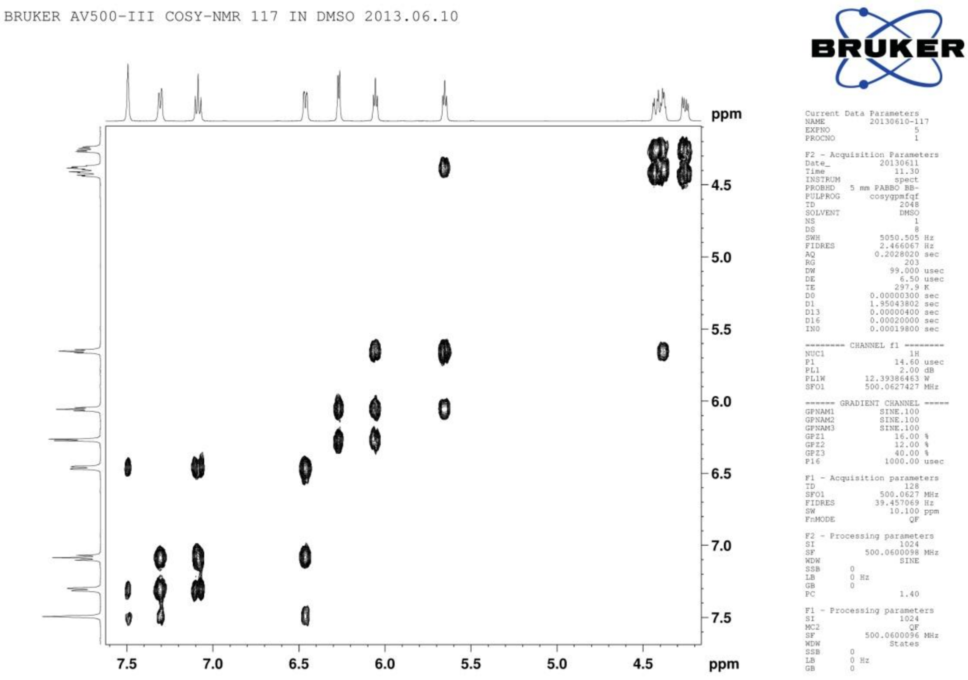

Fig. S1-7 HSQC NMR spectrum of WS010117 (500 MHz, DMSO, 25 ℃).

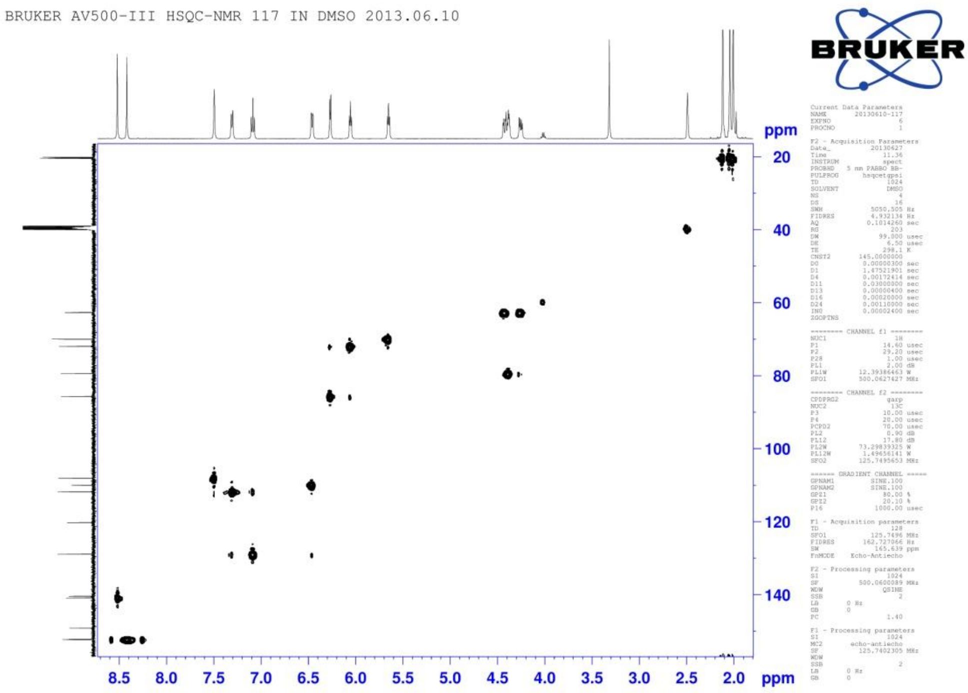

Fig. S1-8 HSQC NMR spectrum of WS010117 (500 MHz, DMSO, 25 ℃).

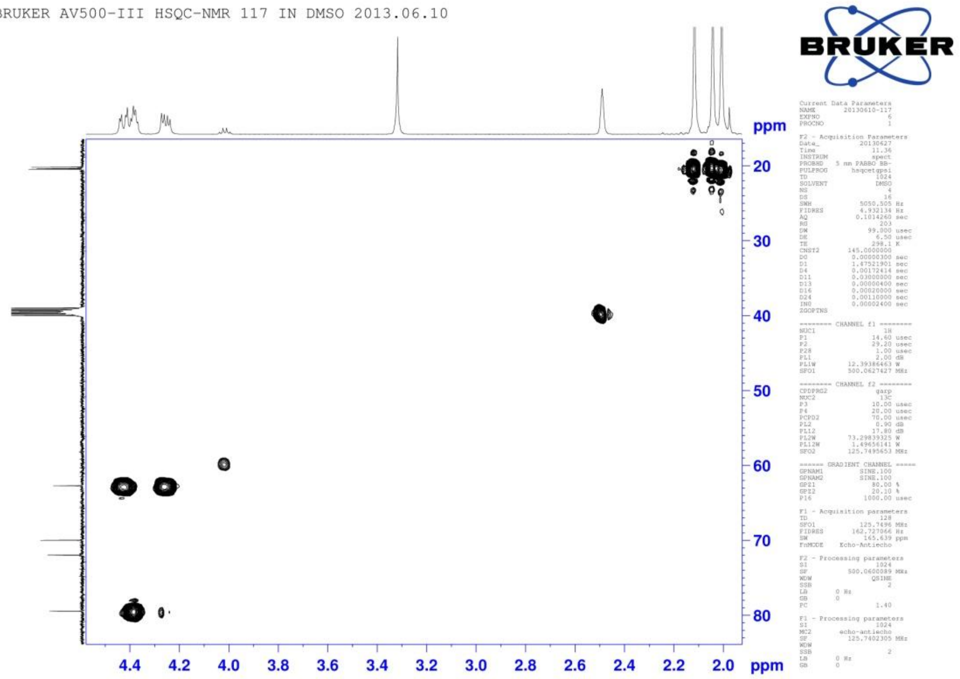

Fig. S1-9 HSQC NMR spectrum of WS010117 (500 MHz, DMSO, 25 ℃).

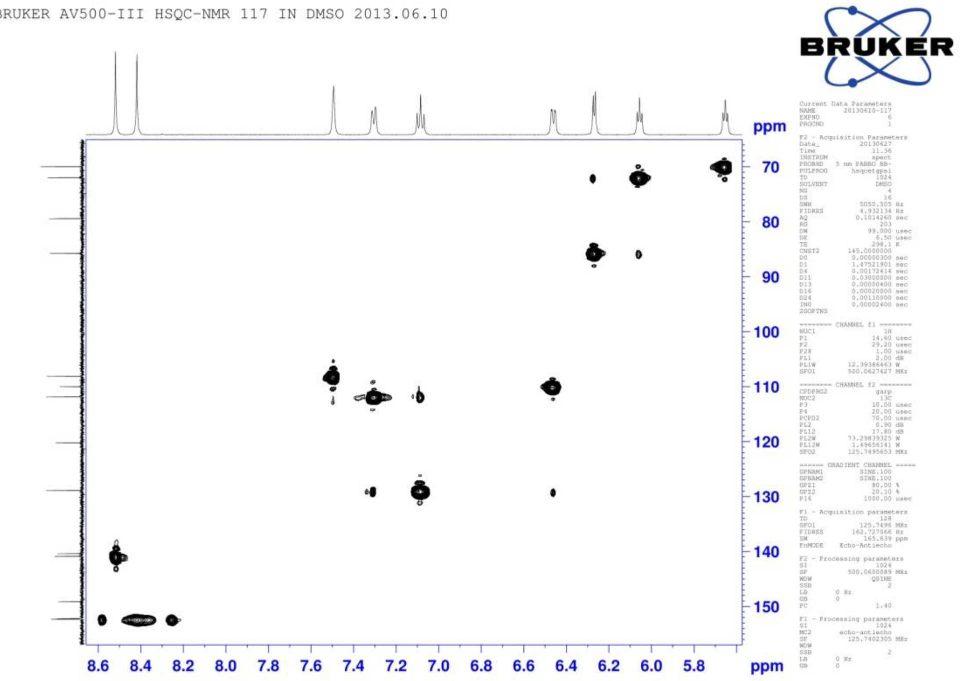

Fig. S1-10 HMBC NMR spectrum of WS010117 (500 MHz, DMSO, 25 ℃).

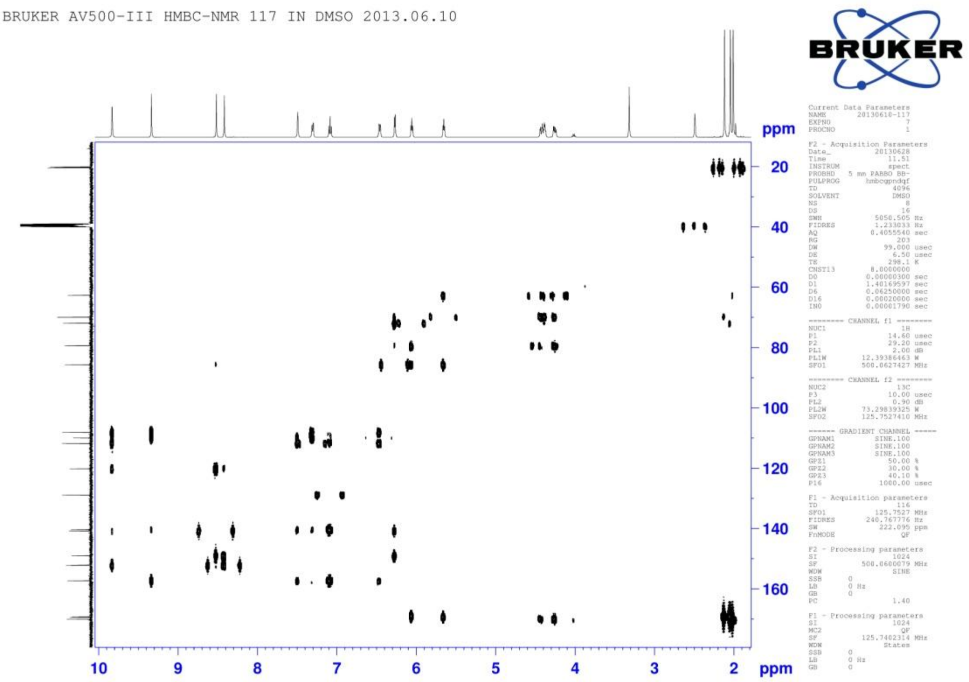

Fig. S1-11 HMBC NMR spectrum of WS010117 (500 MHz, DMSO, 25 ℃).

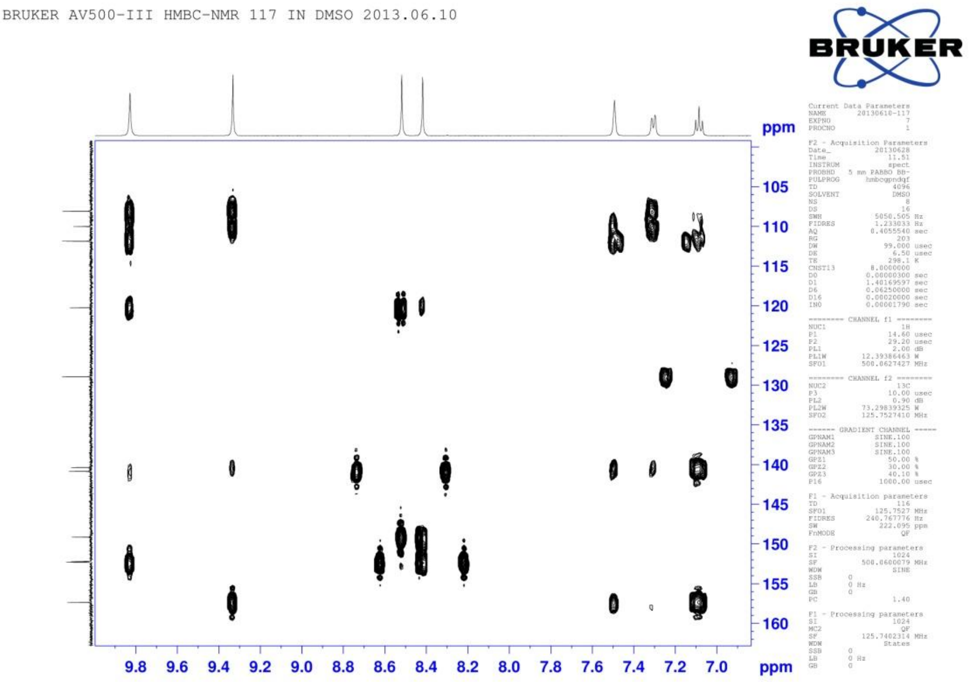

Fig. S1-12 HMBC NMR spectrum of WS010117 (500 MHz, DMSO, 25 ℃).

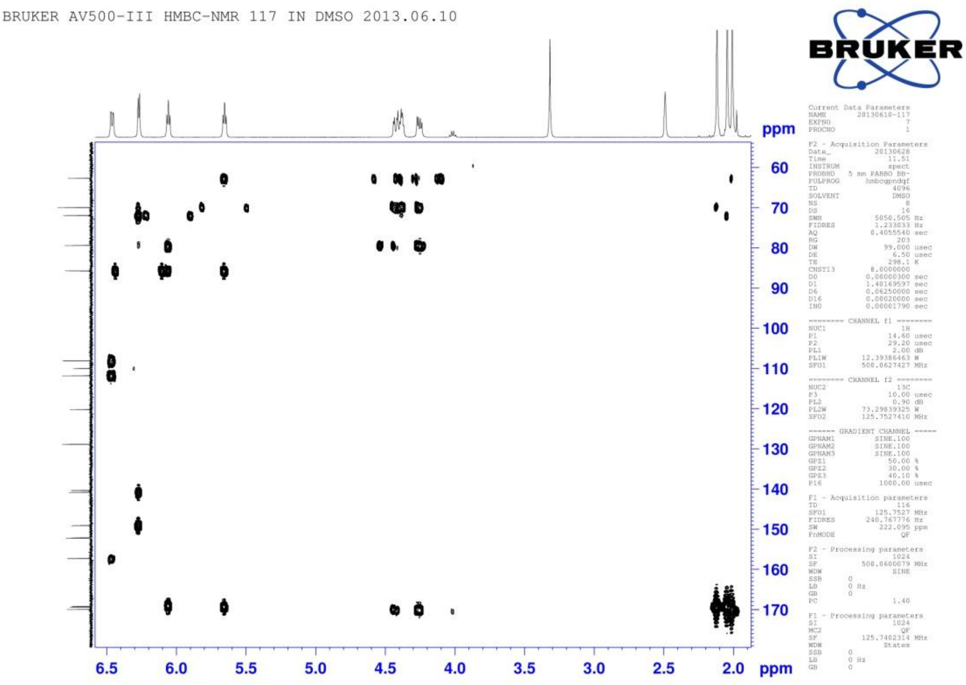

Supplement: S1 File — (PDF) [file pone.0127583.s001.pdf]
