# Supplementary material for: Structure Elucidation of the Metabolites of 2', 3', 5'-Tri-O-Acetyl-N 6-(3-Hydroxyphenyl) Adenosine in Rat Urine by HPLC-DAD, ESI-MS and Off-Line Microprobe NMR
Source: PLoS One. 2015 Jun 1;10(6):e0127583. doi: 10.1371/journal.pone.0127583 (PMC4451981; doi:10.1371/journal.pone.0127583)

S2 File. The NMR spectra of M2.

Fig. S2-1 ^{1}H NMR spectrum of M2 (500 MHz, DMSO, 25 ℃).

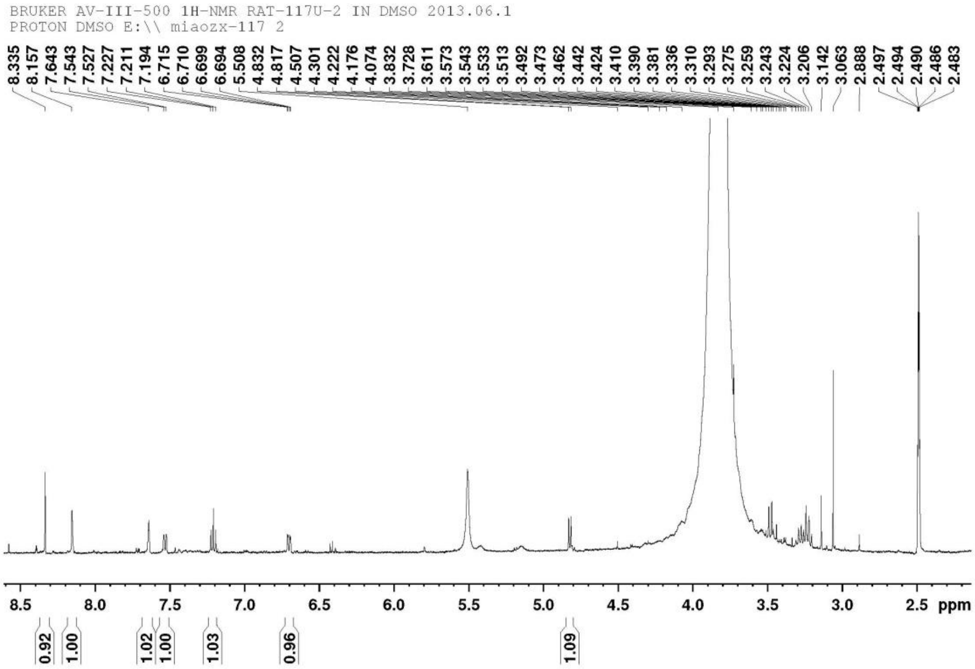

Fig. S2-2 ^{1}H NMR spectrum of M2 (500 MHz, DMSO, 25 ℃).

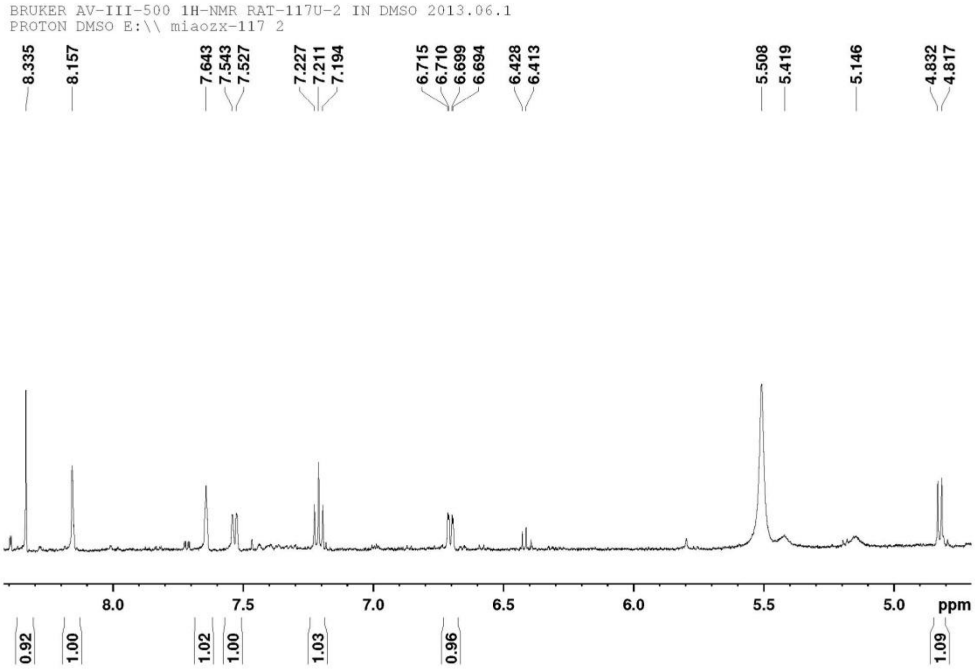

Fig. S2-3 COSY NMR spectrum of M2 (500 MHz, DMSO, 25 ℃).

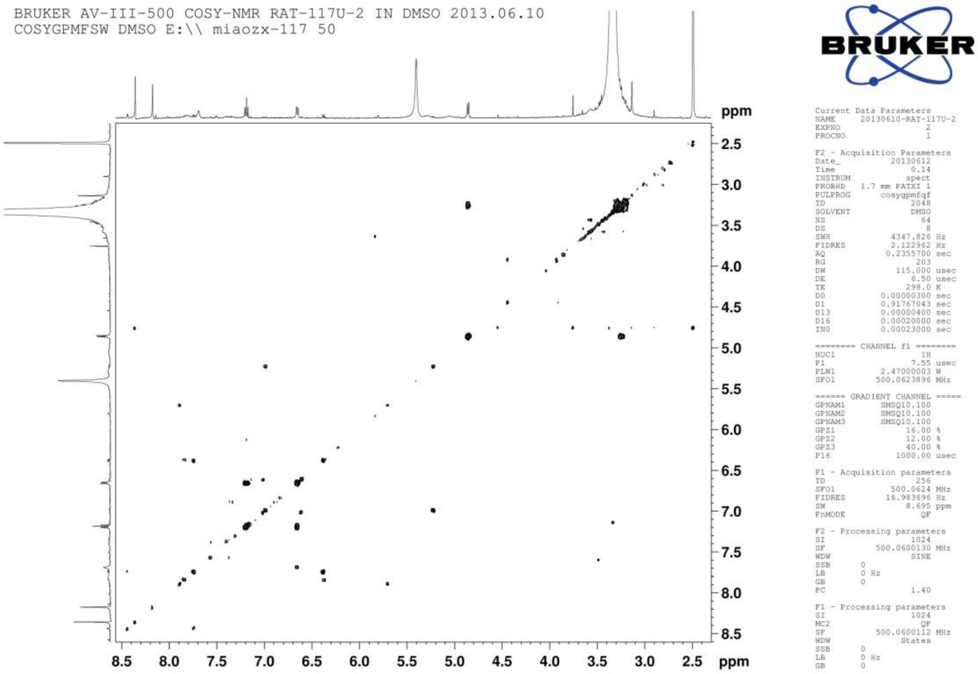

Fig. S2-4 HSQC NMR spectrum of M2 (500 MHz, DMSO, 25 ℃).

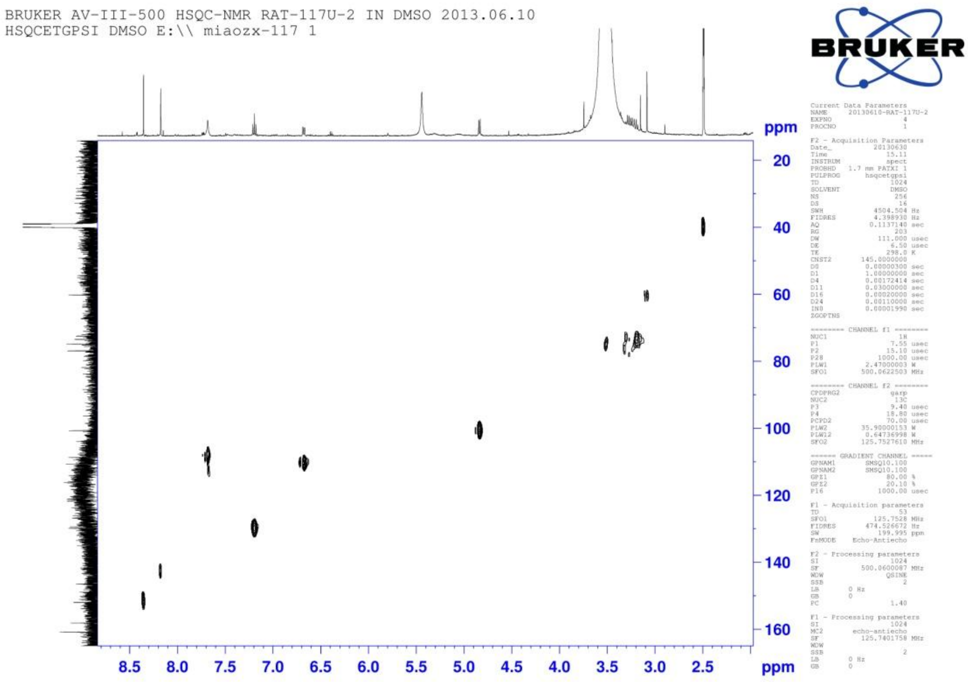

Fig. S2-5 HMBC NMR spectrum of M2 (500 MHz, DMSO, 25 ℃).

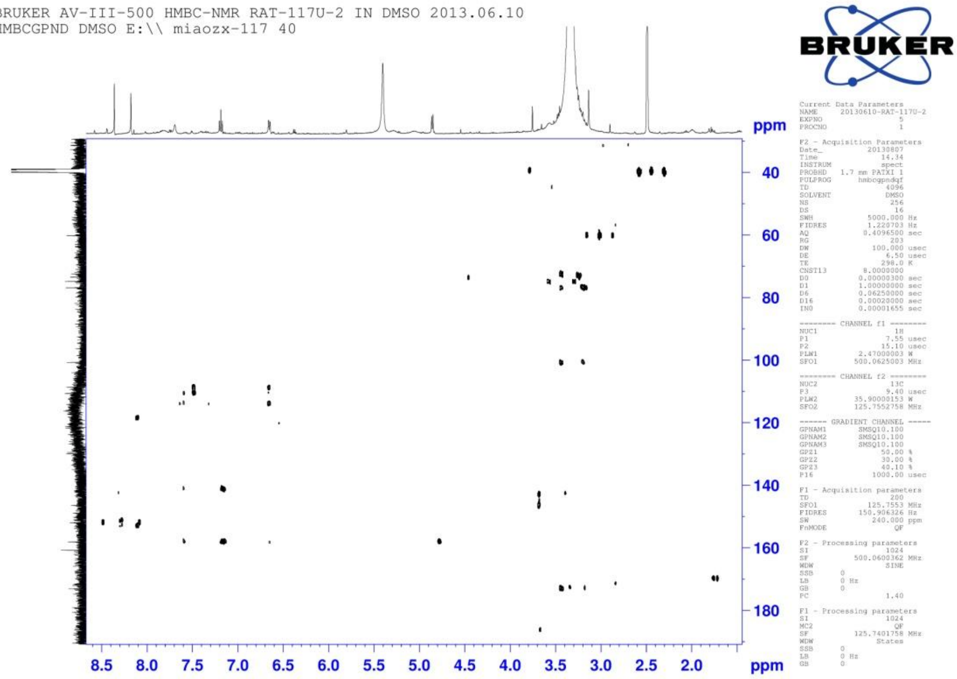

Fig. S2-6 HMBC NMR spectrum of M2 (500 MHz, DMSO, 25 ℃).

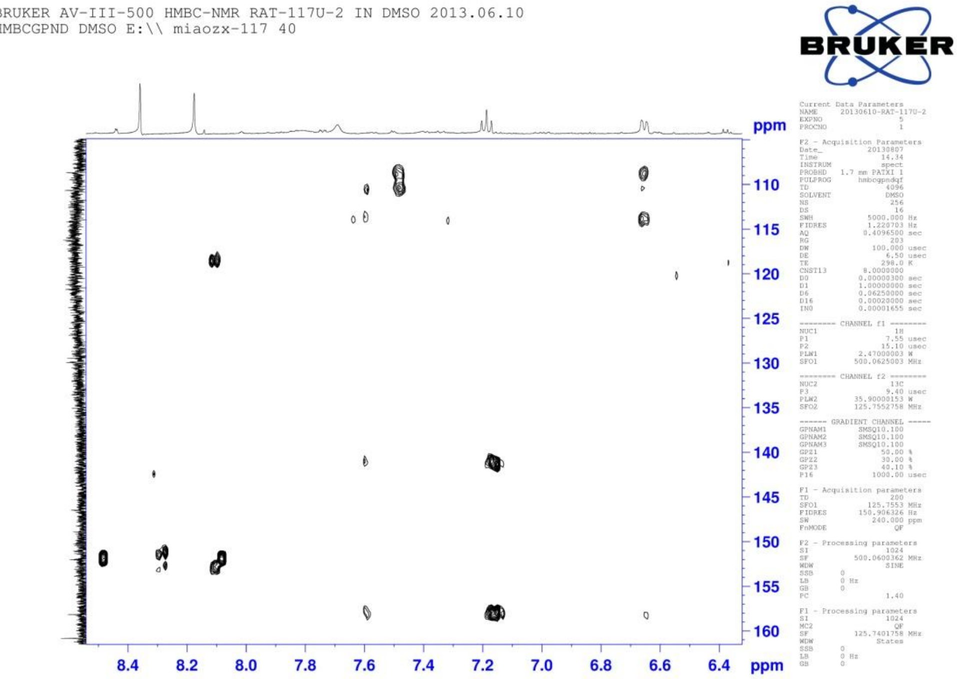

Fig. S2-7 HMBC NMR spectrum of M2 (500 MHz, DMSO, 25 ℃).

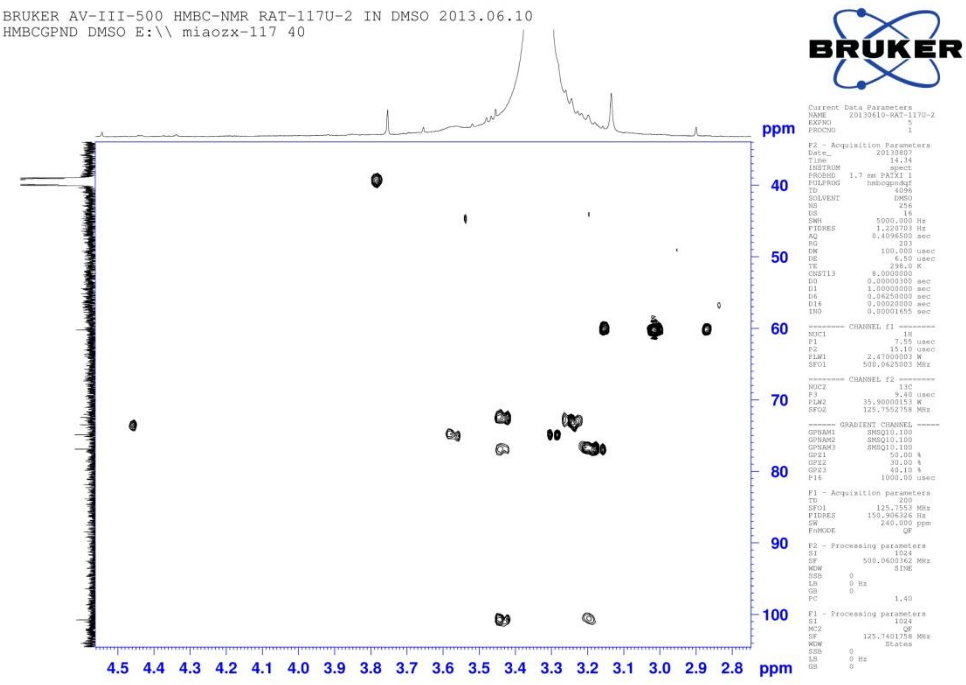

Fig. S2-8 HMBC NMR spectrum of M2 (500 MHz, DMSO, 25 ℃).

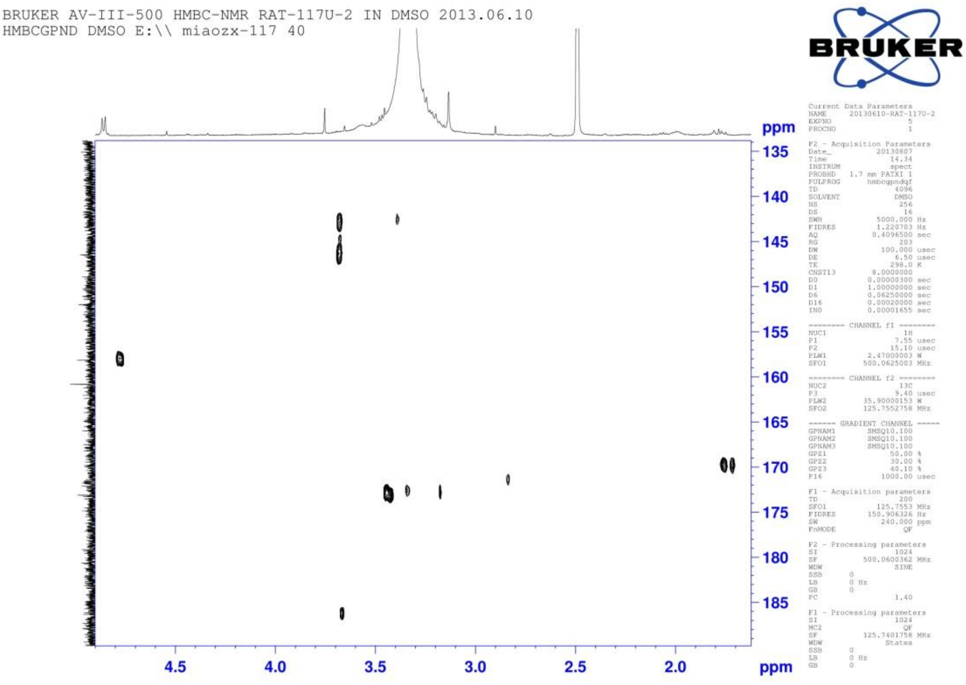

Supplement: S2 File — (PDF) [file pone.0127583.s002.pdf]
