# Supplementary material for: Structure Elucidation of the Metabolites of 2', 3', 5'-Tri-O-Acetyl-N 6-(3-Hydroxyphenyl) Adenosine in Rat Urine by HPLC-DAD, ESI-MS and Off-Line Microprobe NMR
Source: PLoS One. 2015 Jun 1;10(6):e0127583. doi: 10.1371/journal.pone.0127583 (PMC4451981; doi:10.1371/journal.pone.0127583)

S4 File. The NMR spectra of M4.

Fig. S4-1 ^{1}H NMR spectrum of M4 (500 MHz, DMSO, 25 ℃).

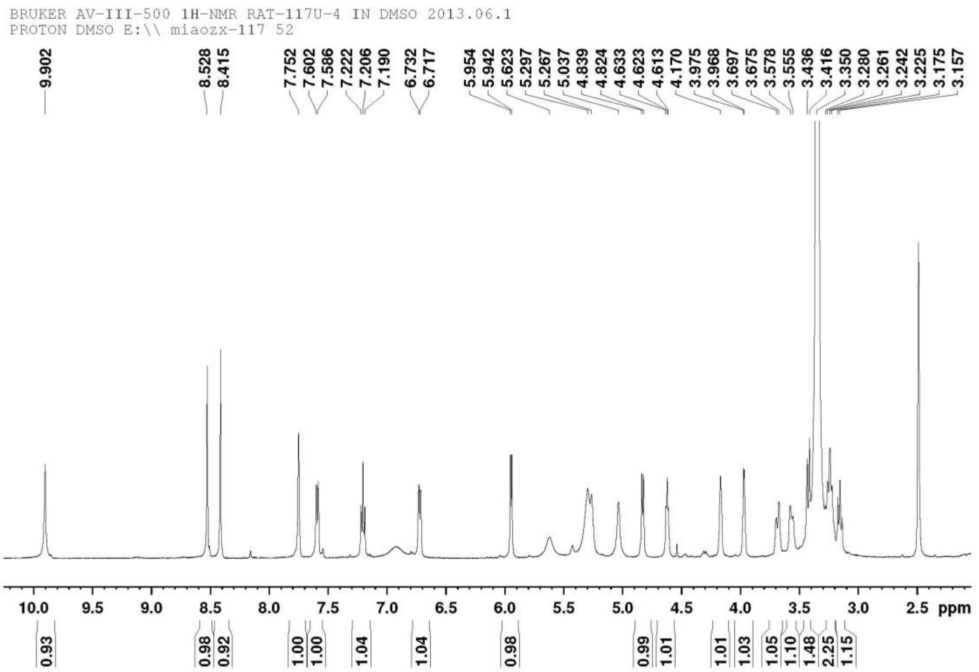

Fig. S4-2 ^{1}H NMR spectrum of M4 (500 MHz, DMSO, 25 ℃).

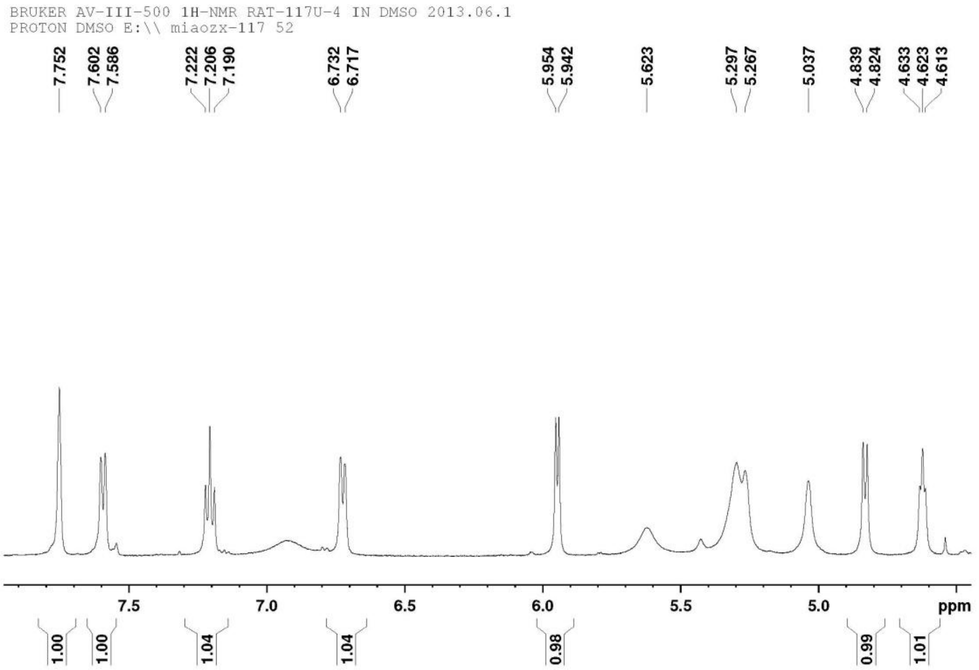

Fig. S4-3 ^{1}H NMR spectrum of M4 (500 MHz, DMSO, 25 ℃).

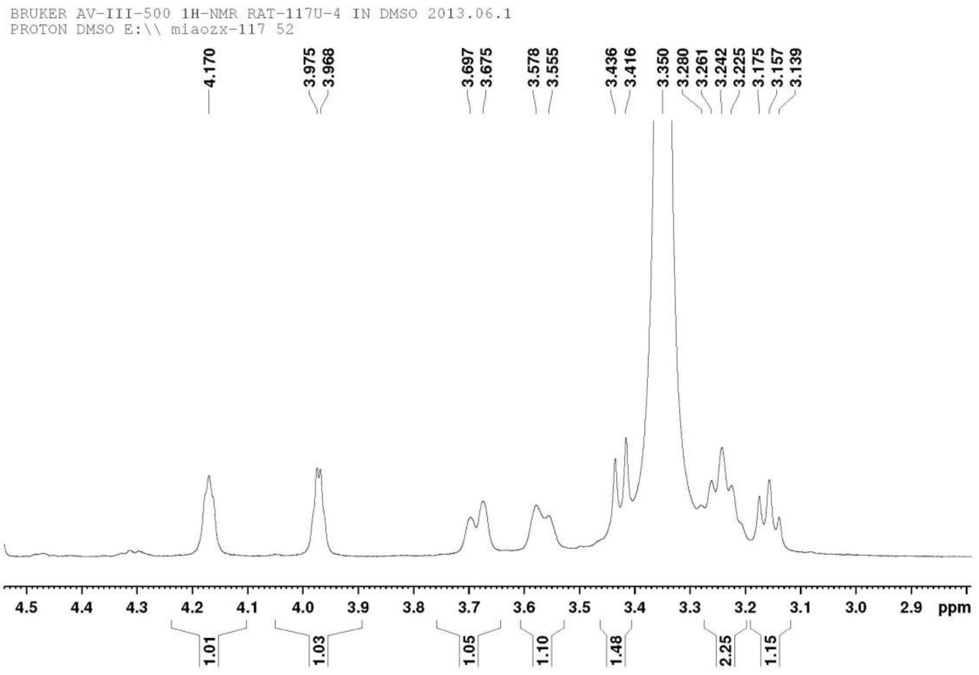

Fig. S4-4 ^{13}C NMR spectrum of M4 (500 MHz, DMSO, 25 ℃).

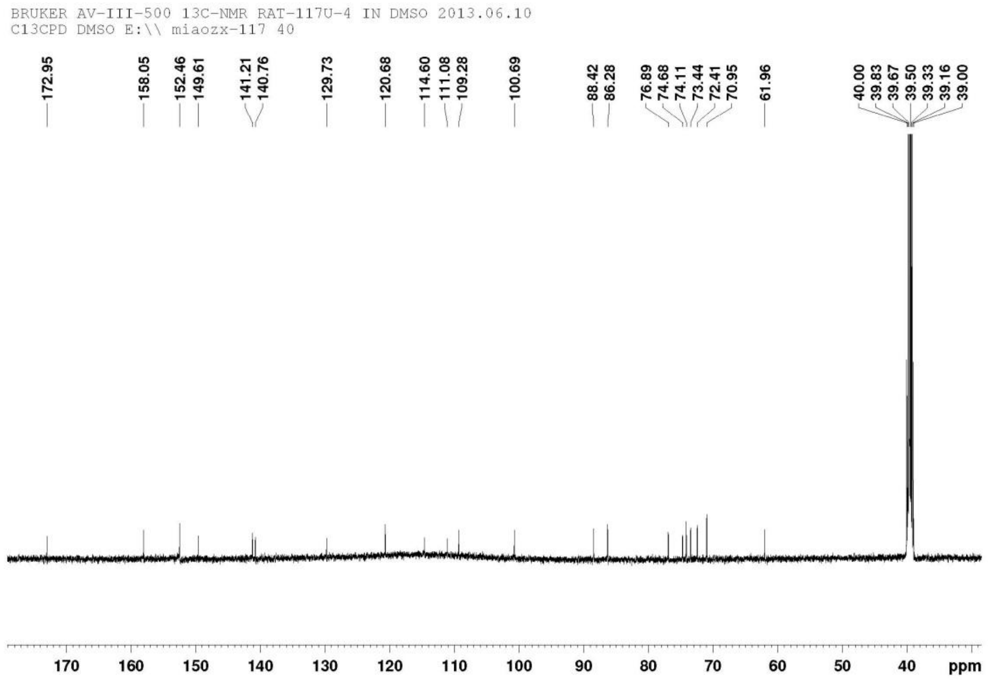

Fig. S4-5 COSY NMR spectrum of M4 (500 MHz, DMSO, 25 ℃).

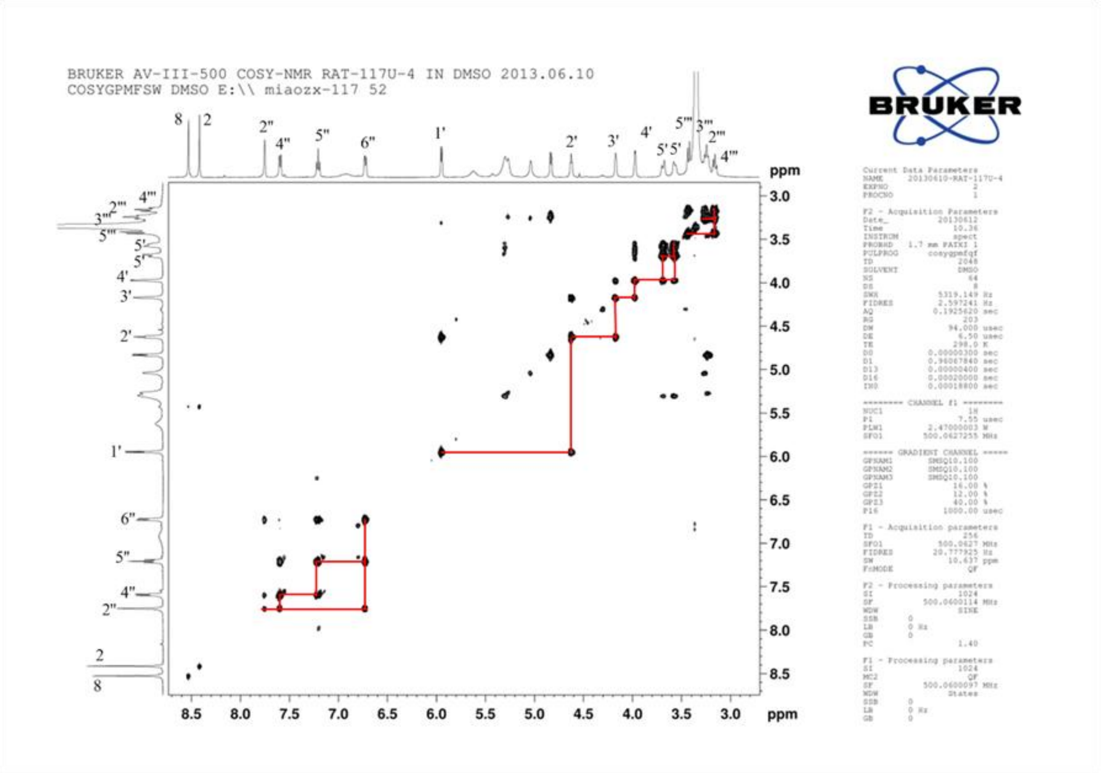

Fig. S4-6 COSY NMR spectrum of M4 (500 MHz, DMSO, 25 ℃).

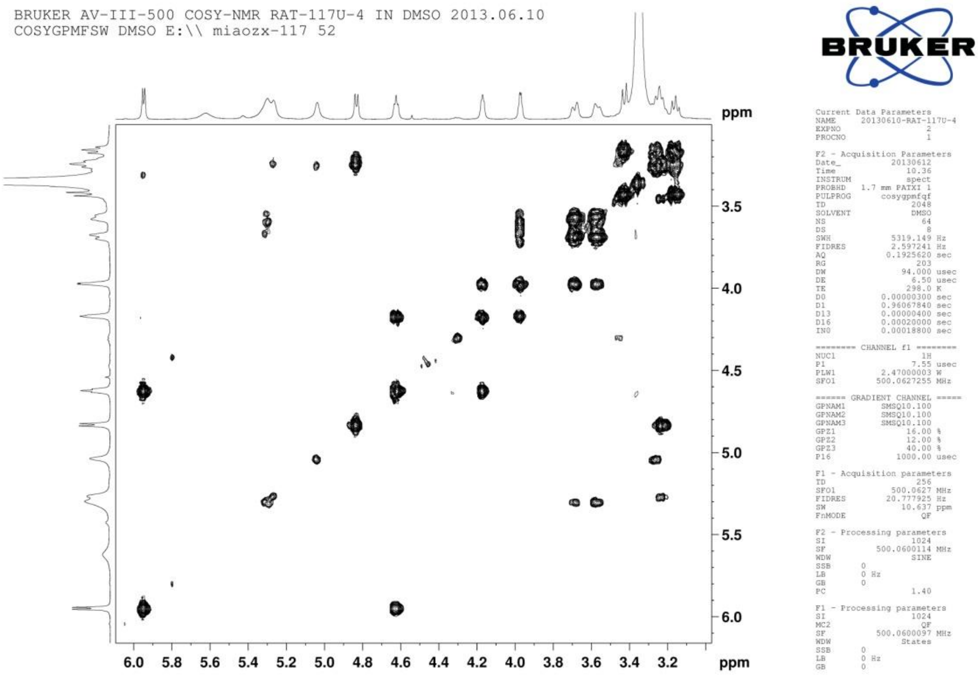

Fig. S4-7 HSQC NMR spectrum of M4 (500 MHz, DMSO, 25 ℃).

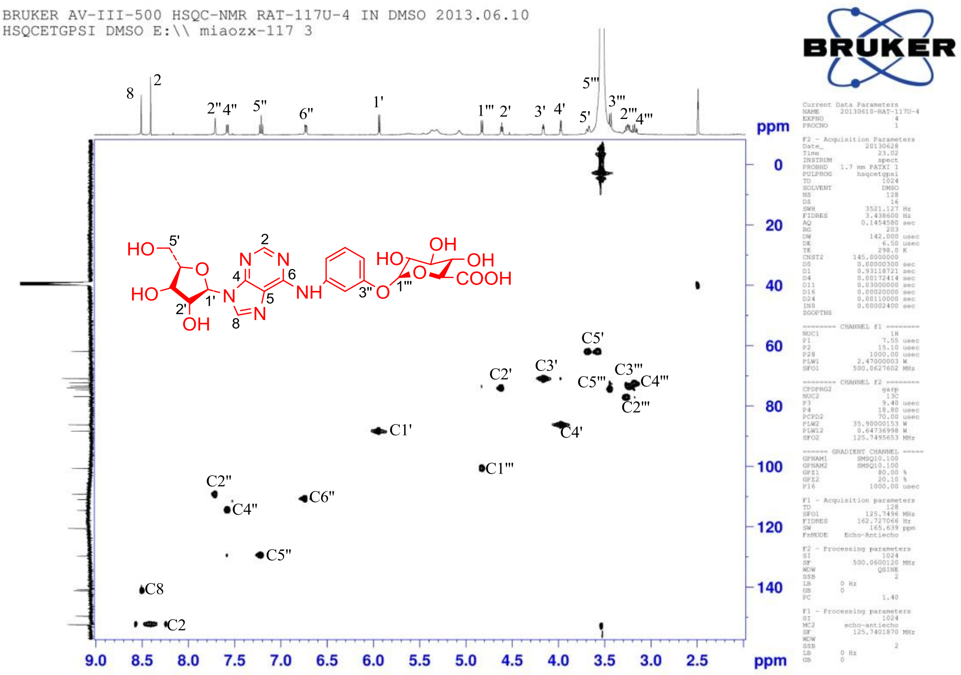

Fig. S4-8 HSQC NMR spectrum of M4 (500 MHz, DMSO, 25 ℃).

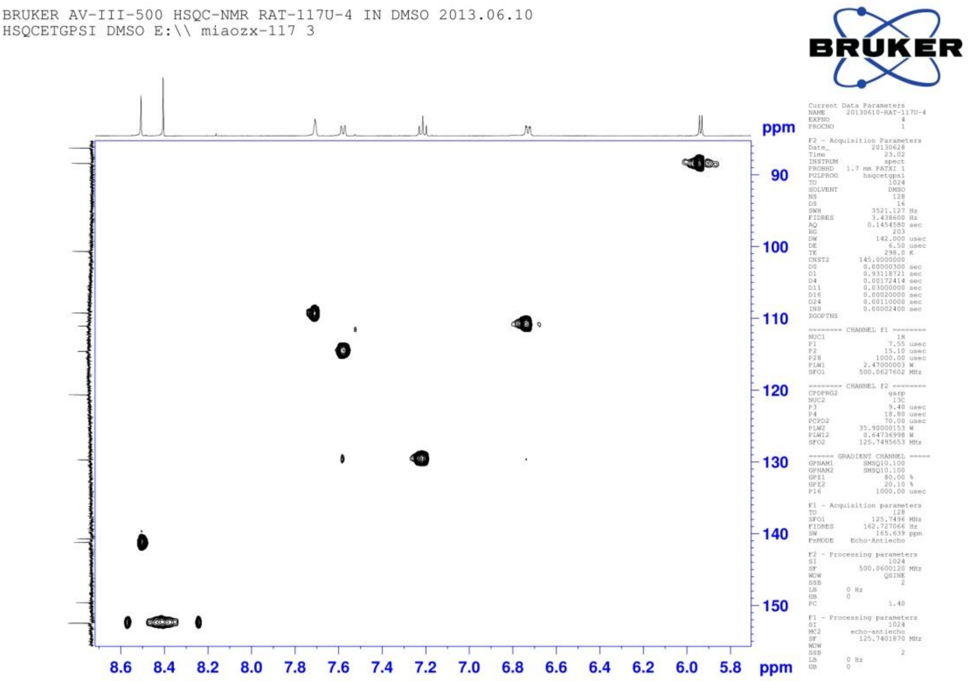

Fig. S4-9 HSQC NMR spectrum of M4 (500 MHz, DMSO, 25 ℃).

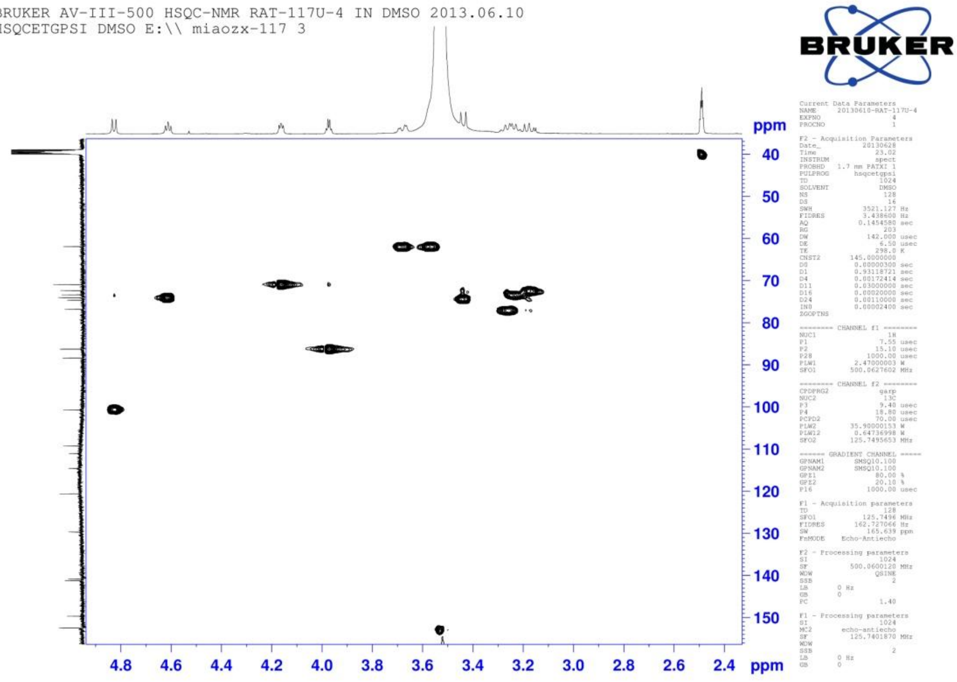

Fig. S4-10 HMBC NMR spectrum of M4 (500 MHz, DMSO, 25 ℃).

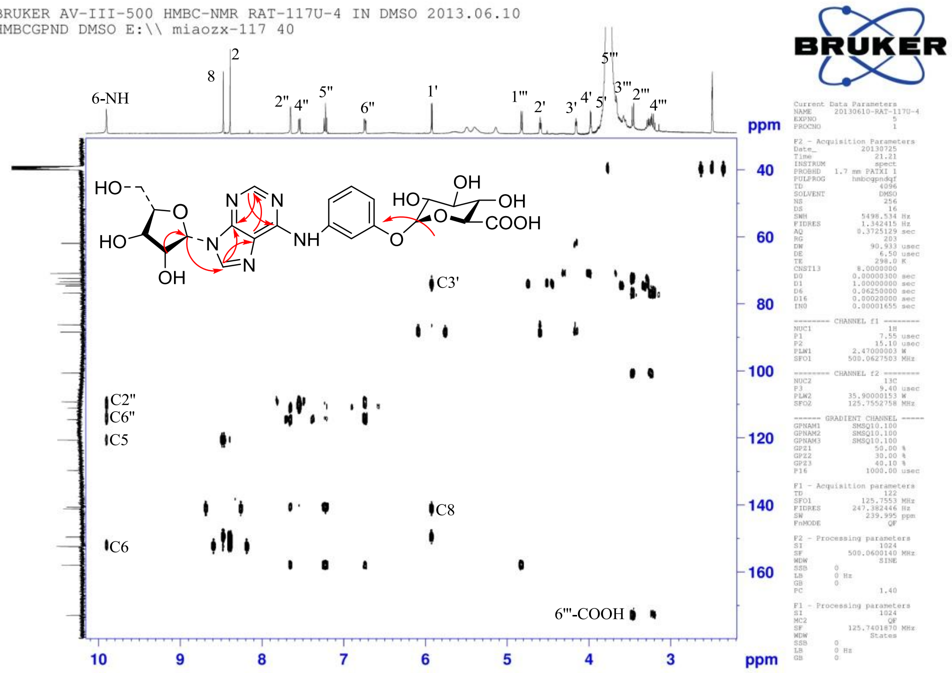

Fig. S4-11 HMBC NMR spectrum of M4 (500 MHz, DMSO, 25 ℃).

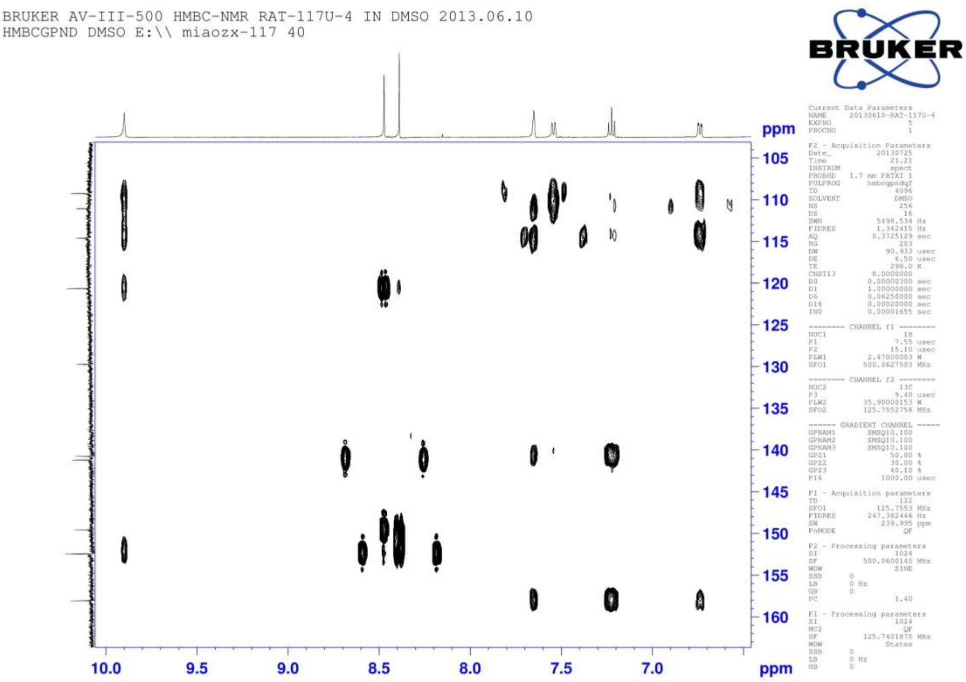

Fig. S4-12 HMBC NMR spectrum of M4 (500 MHz, DMSO, 25 ℃).

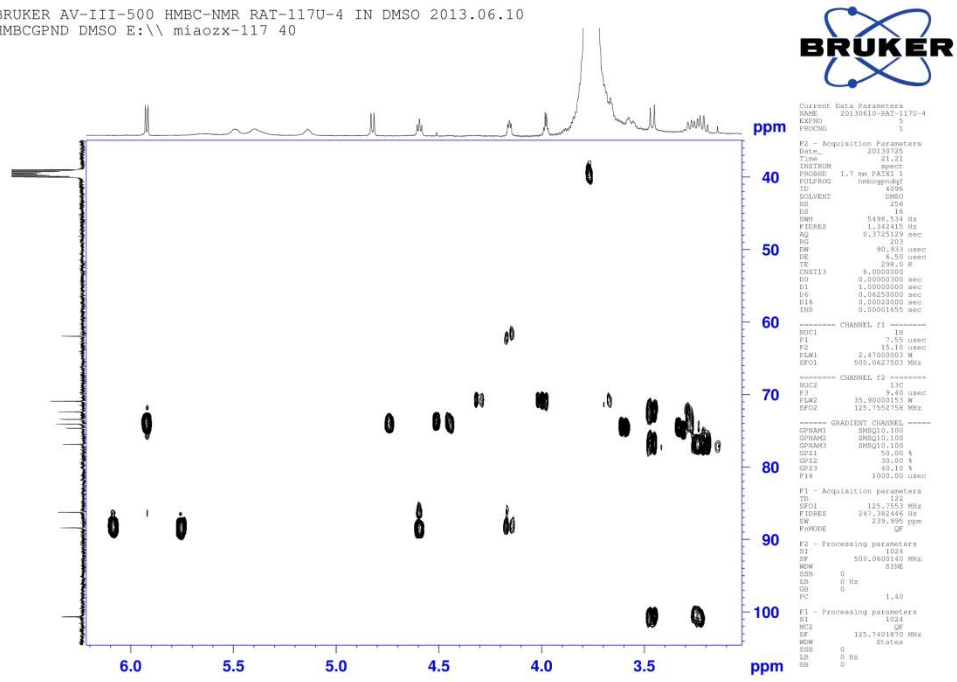

Fig. S4-13 HMBC NMR spectrum of M4 (500 MHz, DMSO, 25 ℃).

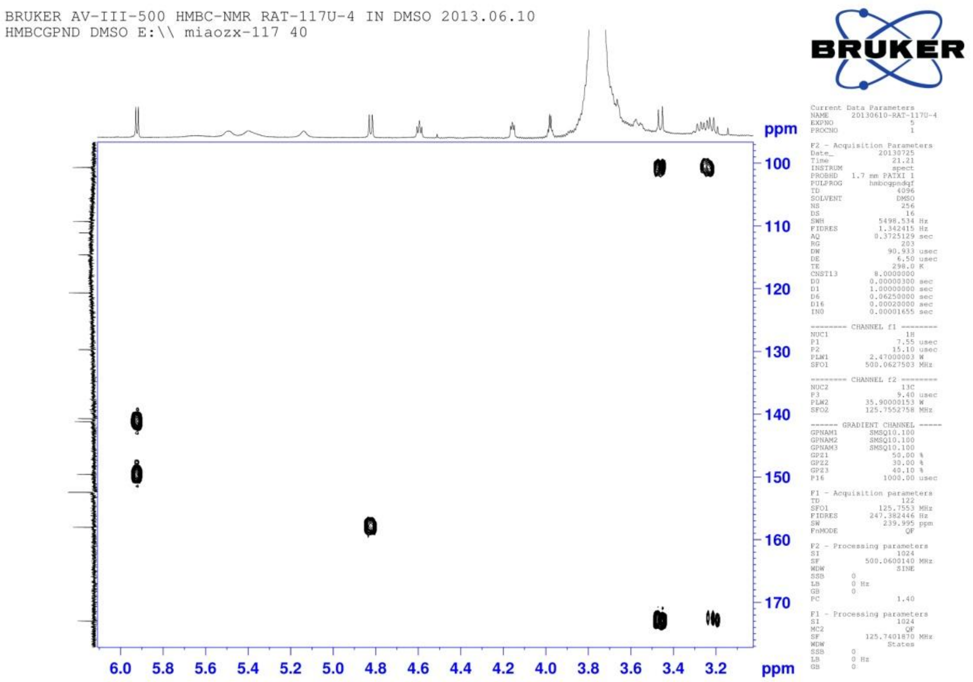

Supplement: S4 File — (PDF) [file pone.0127583.s004.pdf]
